# Divergent Whole Brain Projections from the Ventral Midbrain in Macaques

**DOI:** 10.1093/cercor/bhaa399

**Published:** 2021-02-09

**Authors:** Muhammad Zubair, Sjoerd R Murris, Kaoru Isa, Hirotaka Onoe, Yoshinori Koshimizu, Kenta Kobayashi, Wim Vanduffel, Tadashi Isa

**Affiliations:** Laboratory of Neuro- and Psychophysiology, Department of Neurosciences, KU Leuven Medical School, Leuven 3000, Belgium; Leuven Brain Institute, Leuven 3000, Belgium; Department of Neuroscience, Graduate School of Medicine, Kyoto University, Kyoto 606-8501, Japan; Laboratory of Neuro- and Psychophysiology, Department of Neurosciences, KU Leuven Medical School, Leuven 3000, Belgium; Leuven Brain Institute, Leuven 3000, Belgium; Department of Neuroscience, Graduate School of Medicine, Kyoto University, Kyoto 606-8501, Japan; Human Brain Research Center, Graduate School of Medicine, Kyoto University, Kyoto 606-8507, Japan; Department of Neuroscience, Graduate School of Medicine, Kyoto University, Kyoto 606-8501, Japan; Section of Viral Vector Development, National Institute for Physiological Sciences, Okazaki, 444-8585, Japan; Laboratory of Neuro- and Psychophysiology, Department of Neurosciences, KU Leuven Medical School, Leuven 3000, Belgium; Leuven Brain Institute, Leuven 3000, Belgium; Athinoula A. Martinos Center for Biomedical Imaging, Massachusetts General Hospital, Charlestown, MA 02129, USA; Department of Radiology, Harvard Medical School, Boston, MA 02144, USA; Department of Neuroscience, Graduate School of Medicine, Kyoto University, Kyoto 606-8501, Japan; Human Brain Research Center, Graduate School of Medicine, Kyoto University, Kyoto 606-8507, Japan; Institute for the Advanced Study of Human Biology (WPI-ASHBi), Kyoto University, Kyoto 606-8501, Japan

**Keywords:** AAV, anterograde projection, dopamine, immunohistochemistry, *Macaca fuscata*, VTA

## Abstract

To understand the connectome of the axonal arborizations of dopaminergic midbrain neurons, we investigated the anterograde spread of highly sensitive viral tracers injected into the ventral tegmental area (VTA) and adjacent areas in 3 macaques. In 2 monkeys, injections were centered on the lateral VTA with some spread into the substantia nigra, while in one animal the injection targeted the medial VTA with partial spread into the ventro-medial thalamus. Double-labeling with antibodies against transduced fluorescent proteins (FPs) and tyrosine hydroxylase indicated that substantial portions of transduced midbrain neurons were dopaminergic. Interestingly, cortical terminals were found either homogeneously in molecular layer I, or more heterogeneously, sometimes forming patches, in the deeper laminae II–VI. In the animals with injections in lateral VTA, terminals were most dense in somatomotor cortex and the striatum. In contrast, when the medial VTA was transduced, dense terminals were found in dorsal prefrontal and temporal cortices, while projections to striatum were sparse. In all monkeys, orbitofrontal and occipito-parietal cortex received strong and weak innervation, respectively. Thus, the dopaminergic ventral midbrain sends heterogeneous projections throughout the brain. Furthermore, our results suggest the existence of subgroups in meso-dopaminergic neurons depending on their location in the primate ventral midbrain.

## Introduction

The mammalian midbrain houses several nuclei that contain predominantly dopaminergic cells. These mesencephalic clusters are classically divided into 3 major subgroups: the ventral tegmental area (VTA or A10), the substantia nigra (SN or A9: which includes both the pars compacta (SNc) and pars reticulata (SNr)) and the retrorubral field (RRF or A8) (Dahlström 1964). Individual axons originating from dopaminergic cell bodies within these midbrain nuclei branch into many cortical and subcortical target areas (Matsuda et al. 2009; Aransay et al. 2015). In addition, the omnipresence of dopamine (DA) receptors and terminals throughout the brain in rodents (Weiner et al. 1991), nonhuman primates (Brown et al. 1979; Berger et al. 1988; Lidow et al. 1991) and humans (Gaspar et al. 1989) emphasizes the importance of these midbrain subgroups, which are the nearly exclusive source of DA within our brain.

A wide range of functions is associated with dopamine, such as reward and reward prediction error signaling (Schultz 2015), working memory (Sawaguchi and Goldman-Rakic 1991; Gamo et al. 2010), inhibitory control (Chamberlain et al. 2006), motor behavior (Kunori et al. 2014), attention (Noudoost and Moore 2011) and motivation and effort (Walton and Bouret 2019; Vancraeyenest et al. 2020). In attempts to ascertain the functional contribution of the midbrain to cortical and subcortical processing, previous studies combined electrical or optogenetic stimulation of the VTA in macaque monkeys with concurrent physiological (including functional magnetic resonance imaging, fMRI) and/or behavioral measures. Stimulation of this structure has a reinforcing effect within both instrumental and Pavlovian conditioning paradigms (Arsenault et al. 2014; Stauffer et al. 2016). Furthermore, concurrent brain-wide fMRI activation induced by VTA stimulation was detected in a network of mainly reward-related areas in monkeys (Arsenault *et al.* 2014). Comparable rodent studies (i.e., through optogenetics) have found, however, that the fMRI effects of midbrain photostimulation are more widespread than expected and potentially extend beyond this canonical reward network (Decot et al. 2017; Lohani et al. 2017). These seemingly incompatible findings could be the result of the testing conditions, such as stimulation parameters (amplitude, frequency, duration etc.) and the state of the animal (Murris et al. 2020). Perhaps the potentially largest contributor, however, to these heterogeneous functional and behavioral effects evoked by stimulating the DA system relates to the affected nuclei and/or cell-types that are specifically activated within the midbrain. Recent rodent studies linking a conditioned visual stimulus to photostimulation of the more dorsal lateral midbrain (mostly SNc) lead to an increase in undirected locomotion in the animals, whereas photostimulation of the more ventral medial midbrain (mostly VTA) resulted in more active stimulus-directed learning (Saunders et al. 2018; Keiflin et al. 2019).

To tackle outstanding questions on the multifaceted nature of the DA-system, it is crucial to precisely understand its underlying structural architecture. Thus far, however, despite the pioneering studies on the dopaminergic forebrain projections by Goldman-Rakic and colleagues (Levitt et al. 1984; Lidow *et al.* 1991), the efferent connectivity profile of the dopaminergic midbrain has remained under-investigated in primates. The injection of retrograde tracers has revealed strong projections from the midbrain to a handful of regions including the prefrontal cortex (PFC) (Williams and Goldman-Rakic 1998; Frankle et al. 2006), the striatum (Haber et al. 2000) and sparse projections to higher visual area (Gattass et al. 2014) in primates. Still, the caveat of retrograde tracers is that they do not permit a full brain exploration and the density of innervation in individual cortical laminae is impossible to assess using retrograde tracing.

The recent advent and development of viral vectors within neuroscience allows for improved anterograde tracing of neural projections (Davidsson et al. 2019). Here, we took advantage of these technological developments and injected the midbrain of 3 monkeys, with adeno-associated virus (AAV). We found that projections originating from the VTA (with some inclusion of neighboring nuclei) cover the entire brain, but with clearly heterogeneous patterns of regions with high- and low-density innervation, and with profound differences across layers. There are hotspots with dense projections and patchy expressions of reporter genes in regions such as the striatum and orbitofrontal cortex (OFC). We also found obvious differences in the projection patterns across subjects, likely due to small variations in midbrain injection sites.

## Materials And Methods

### Subjects

Three female macaque monkeys (*Macaca fuscata*; M1: 4.2 kg, 4 years old; M2: 6.7 kg, 7 years old; M3: 9.4 kg, 9 years old) were used in the present study (Supplementary Material S1). Animal care and experimental procedures were performed in accordance with the ILAR’s Guide for the Care and Use of Laboratory Animals and were approved by the committee for animal experiments at the Graduate School of Medicine in Kyoto University, Japan. Monkeys are fed daily with standard primate chow, water and supplemented with fruits.

### Viral Vector Preparation

AAV vectors were packaged as previously described (Kobayashi et al. 2016). Briefly, the packaging (pAAV2.1 or pAAV-DJ and pHelper) and transfer plasmids (pAAV-CAG-DsRed2 and pAAV-CAG-hChR2(H134R)-tdTomato) were transfected into HEK293T cells. The pAAV2.1 was prepared by mixing pAAV-RC1 and pAAV-RC2 plasmids; the weight ratio of pAAV-RC1 to pAAV-RC2 is 1:9. After incubation, harvested cells were lysed and purified by serial ultracentrifugation with cesium chloride. The purified particles were dialyzed with 0.001% Pluronic-F68 Solution (Sigma-Aldrich; St. Louis, MO USA) in phosphate-buffered saline (PBS), and then concentrated through ultrafiltration. The copy number of the viral genome (vg) was determined using a TaqMan Universal Master Mix II (Applied Biosystems; Foster City, CA USA). The present experiments were conducted to co-examine the expression of ChR2 in the axons originating in the VTA for potential future optogenetic stimulation experiments. However, the present animals were not used for the actual optogenetic experiments.

### MRI/CT-Guided Targeting and Injection Procedures

For the precise injection of AAV vectors into the VTA, we performed magnetic resonance imaging (MRI) and computed tomographic (CT) imaging by using a 3-Tesla MRI scanner (Verio, Siemens, USA) and a 320-detector-row CT scanner (Aquilion ONE, Toshiba Medical Systems Corporation, Japan), respectively. For all surgeries, anesthesia of the monkeys was initially induced by intramuscular injections of xylazine hydrochloride (2 mg/kg), ketamine hydrochloride (5 mg/kg) and atropine sulfate (0.04 mg/kg), and subsequently maintained through the inhalation of isoflurane (1.0–1.5%) from a ventilator. Vital functions (i.e., heart rate, respiration rate, oxygen saturation, body temperature, and end-tidal carbon dioxide) were monitored throughout surgeries. First, the MR and CT images were obtained while the animals’ head was fixed in a custom-designed acrylic MRI-compatible stereotactic apparatus under anesthesia. A custom-built 8-channel birdcage radiofrequency coil with a 10 cm inner diameter (Takashima Seisakusho Co., Ltd, Japan) was used for MRI. High-resolution 3D T1- and T2-weighted images were obtained through a 3D fast spin-echo sequence with 2 echoes. The separate CT and MR images were overlaid by applying a linear, rigid coregistration algorithm (PMOD Technologies, Zürich, Switzerland), in which the MR image was aligned on the subject’s skull visible on the CT image. The merged image could subsequently be used to determine the exact positioning of a chamber on the skull, which allowed to target the VTA. This chamber could hold a stereotactic plastic grid for fixing an injection needle, with holes at 1 mm intervals (6-YGD-D1, Crist, USA). For monkeys 1 and 2, 2 chambers were tilted back 30 degrees in the sagittal plane and 25 degrees in the coronal plane for each hemisphere. For M3, the first monkey which we injected in this study, a single chamber was positioned vertically on the skull above the VTA. In this subject, we observed dorsal leakage of the viral vector. Therefore, we modified the injection methods in M1 and M2 as described below.

A second series of MRI/CT images was acquired under anesthesia, but after chamber implantation and with the grid in place, to determine the exact position of the needle within the grid and depth of the injection along the track. To verify and visualize the exact injection site for the VTA of each monkey, a craniotomy was made under the grid and a gadolinium solution (OMNISCAN, Daiichi-Sankyo, Japan) at a concentration of 0.02 μmol/μL was injected into the intended position (rate: 0.1 μL/min, total volume: 0.5 μL). A subsequent acquisition of a T1- weighted image following the injection revealed the spread of the contrast agent (Supplementary Material S2) and therefore gave us a good indication of the center of mass of the injections.

Exact specifications of the viral vectors, the injections and the animals that were used are provided in Supplementary Material S1. After the injection sites relative to the grid were decided, the monkey was anesthetized again and the AAV vectors were injected into the intended location: the VTA. The injection needles were prepared as previously described (Vancraeyenest *et al.* 2020). Briefly, we first inserted the guide tube (40 mm in total length) to locate its tip about 7.25–9.25 mm above the VTA. Then, a Hamilton needle (28G, 140 mm; point style 2; no hub) was attached to a polytetrafluoroethylene tube and connected to another Hamilton syringe (10 μL, Model 701, Cemented NDL, 26sG, 2 in, point style 2) that were jointly attached to a micro-infusion pump (Legato 130 Syringe Pump, KdScientific). By attaching the injection needle to a micromanipulator (Narishige; Tokyo, Japan), we were able to accurately lower the needle to the previously calculated targets within the midbrain through the guide tube. To minimize leakage of the vector, we slowly inserted the needle first to 0.3–0.5 mm below the target site and raised the needle back to the target site to make space below the tip of the needle. The actual injection of the vector was successively conducted after a 5-min interval to allow for stabilization of the tissue after needle insertion. Subsequently, the vector was injected at a rate of 100 nL/min. Following the initial injection, another 5-min interval ensured that the viral vector was absorbed, and then, the needle was slowly lifted. Postoperatively, the animals were treated with intramuscular analgesics (5 mg/kg, ketoprofen) and antibiotics (5 mg/kg Ampicillin) and kept under quarantine for at least 7days.

### Histological Processing

Approximately 40 days after the viral vector injections, the animals were deeply anesthetized with ketamine and euthanized through intravenous administration of sodium pentobarbital (35 mg/kg). The animal was then transcardially perfused using 0.05 M PBS followed by 4% paraformaldehyde in 0.1 M phosphate buffer (PB pH 7.4). The brain was subsequently extracted from the skull, saturated with 10, 20 and 30% sucrose solutions in PB for cryoprotection, and later sectioned into 40 μm coronal slices with a sliding microtome (REM-710; Yamato, Japan) along the stereotactic coordinates from the rostral to caudal end of the cortical tissue including subcortical structures and parts of the cerebellum.

### Anti- RFP Immunohistochemistry through DAB Staining

For immunohistochemistry (IHC) of vector transfected neurons, a first antibody to the DsRed2 or tdTomato, red fluorescent protein (RFP) was used with subsequent enhancement through diaminobenzidine (DAB) staining. All procedures below were performed at room temperature, unless stated otherwise. Every 24th section from anterior to posterior was incubated in a blocking solution of 10% normal goat serum (NGS) in PBS with 0.3% of Triton X-100 (PBS-T). Sections were then washed with PBS-T and incubated in 1:5000 rabbit polyclonal anti-RFP (Rockland Immunochemicals; Gilberstville, PA USA) in 2% NGS/PBS-T solution overnight at 4°C. On the next day, sections were washed in PBS-T and incubated in 1:200 biotinylated goat anti-rabbit IgG (Vector Laboratories; CA, US) in 2% NGS/PBS-T for 2 h. Afterwards, sections were incubated with 1:200 vectastain elite ABC kit (Avidin-Biotin Complex—Vector Laboratories: CA, USA) in PBS-T for 1 h and visualized with DAB containing 1% nickel ammonium sulfate (0.01% DAB, 1.0% nickel ammonium sulfate, 0.0003% H_2_O_2_) in tris-buffered saline. To enable comparison of the staining strength throughout the series of whole-brain sections of each monkey, these reaction conditions were carefully controlled to be consistent. The sections were counterstained with 0.1% Neutral Red (Merck 101 369, Frankfurt, Germany).

### Double Labeling IHC with anti-RFP and anti-TH Antibodies

To check the injected area of AAVs, direct fluorescence of RFP was observed and microphotographs were taken with a fluorescence microscope (Keyence BZ-X800, BZ-X710; Osaka, Japan). Immunofluorescent staining against tyrosine hydroxylase (TH) was conducted to detect dopaminergic neurons and to outline the VTA and SNc. The anti-TH IHC method is described below. (1) To verify anti-TH immunoreactivity of the infected neurons in the midbrain and (2) to investigate anti-TH immunoreactivity of their axons at target areas, we applied IHC against the RFP to enhance the relatively weak signal in addition to the anti-TH IHC. Sections were incubated with monoclonal mouse anti-RFP (1:500, MBL, Woburn, MA USA; M155–3) and polyclonal rabbit anti-TH (1:500, Merck Millipore; Burlington, MA USA: AB152) in 5% NGS in PBS/T after blocking incubation. Subsequently, sections were visualized with AF555 Goat anti-mouse IgG (1:200, Abcam; Cambridge, UK: ab150117) for RFP, and AF647 goat anti-rabbit IgG (1: 200, Invitrogen; Carlsbad, CA USA: A-11012) or AF488 goat anti-rabbit IgG (1:200, Invitrogen; A-11008) for TH, respectively.

### Histological Assessment and Data Analysis

To assess the presence of the injected vector, IHC-DAB treated sections (see above) were analyzed with a Keyence inverted microscope. Under a high-resolution (1920 × 1024 dpi) condition, the ×4 magnification was sufficient to clearly visualize stained axons at the projection sites. A complete picture of each brain section was acquired through consecutive sampling and subsequent stitching of the magnified images, after which the resulting image was stored offline at its original size. In addition, regions of interest were carefully chosen and images of these representative areas were obtained at more optimal imaging conditions. To assess the areal density of the projections, we compared and equated our sections to comparable schematic coronal slices from a standard monkey atlas (Paxinos et al. 2000). In this detailed analysis, we divided the whole brain into 5 main subdivisions: (1) prefrontal, (2) somatomotor, (3) parieto-occipital, (4) temporal, and (5) subcortical areas. Each of these subdivisions was then subdivided into reference regions from the same atlas. In addition, within each of the cortical regions, we determined the distribution of axon terminals for the different layers based on laminar landmarks visible through counterstaining for neurons (Neutral Red). The layers were split in 3 segments: the molecular layer (lamina I), intermediate layers (laminae II and III), and deeper layers (laminae IV–VI). We considered the subcortical areas as a whole since they lack the typical 6-layered architecture of neocortical areas. The axonal distribution within each area and its layer segments was ranked on a scale within the range of 0–4, from no fiber expression (0) to the highest density of fibers (4). To indicate concentrated expressions of fibers in sparsely innervated regions (so-called patches), we marked the region and layer in question with an asterisk (of which the size denotes its density).

## Results

### Injection Sites

**Figure 1:**
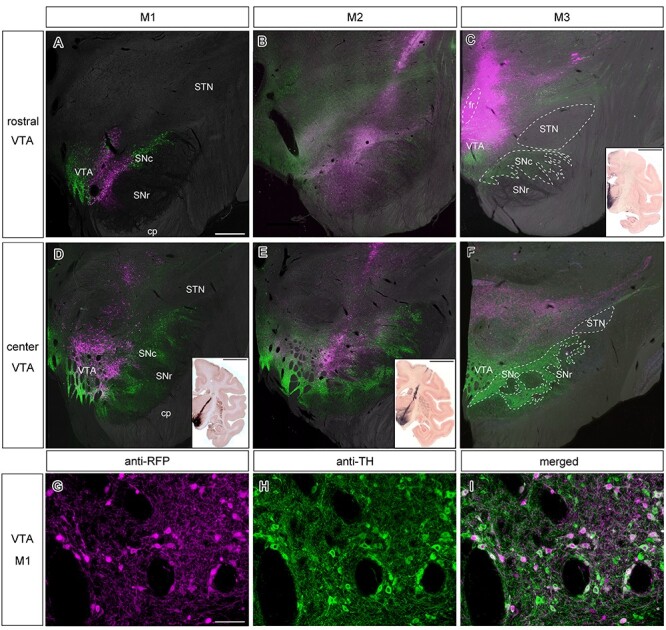
**Viral vector injections targeting the VTA in three monkeys. (A–C)** Photomicrographs of coronal sections in M1–3, revealing the rostral part of the VTA. Superimposition of anti-TH IHC (green), direct RFP fluorescence (magenta) on top of an inverted bright field microscope image. Scale bar in (**A**): 1 mm. The bottom right inset in (**C**) depicts a coronal slice in the vicinity in M3 through enhanced DAB staining, providing a clear overview of the injection area. Scale bar in the inset: 10 mm. **(D–F)** Additional photomicrographs of slightly more posterior coronal sections in M1–3, revealing the central portion of the VTA. We added the borders of the fr, SNc, SNr and STN in (**C**) and (**F**). However, it was not possible to clearly draw the dorsal border of VTA because DA neurons are scattered also in the medial thalamus (Williams and Goldman-Rakic 1998). Staining specifics and the scale bar are the same as in (**A–C**). The more diagonal injection trajectories of M1 and M2 are depicted in the bottom right insets through enhanced DAB staining on coronal sections in the direct vicinity of the magnified images. Scale bar in the inset: 10 mm. **(G–I)** High-magnification view of the VTA in M1, showing double anti-RFP and anti-TH IHC in cell bodies around the injection site. (**G**) anti-RFP IHC, (**H**) anti-TH IHC and (**I**) their merged image. Scale bar: 100 μm. **Abbreviations:** cerebral peduncle (cp), fasciculus retroflexus (fr), substantia nigra pars compacta (SNc), substantia nigra pars reticulata (SNr), subthalamic nucleus (STN).

AAV vectors were injected into the ventral midbrain at multiple sites through a stereotactically positioned plastic grid in 3 animals (see Materials & Methods). The regions infected with the vector injection are shown for the 3 animals in Figure 1A–F. The sections were stained with IHC against tyrosine hydroxylase in which the dopaminergic cell groups of the VTA and SNc (TH^+^: in green) are clearly visible. The injections themselves were visualized through direct fluorescence of RFP which was integrated in the viral vectors (either DsRed2 or tdTomato; RFP^+^ in magenta). In M1, the injection was centered on the middle portion of the VTA along the rostro-caudal axis through laterally inclined injection tracks. The viral vector spread into the medial part of the SNc but spared the medial thalamus **(**Fig. 1A**/D)**. In M2, the injection was centered on the lateral portion of the VTA along the mediolateral axis and the central part of the VTA along the rostrocaudal axis. In addition, the vector spread into the medial two-thirds of the SNc. This injection mostly spared the medial part of the VTA and medial thalamus **(**Fig. 1B**/E)**. In M3, the injection was centered on the dorso-medial, and rostral part of the VTA, excluding the SNc but including a part of the ventro-medial thalamus through vertical injection tracks (Fig. 1C**/F)**. Thus, the injection sites and spread of the injected vector were relatively similar in monkeys M1 and M2, whereas the injected areas overlapped only partially with those in M3. In all 3 animals, the injection spared the RRF (data not shown).

### Cell-type Specificity within Injection Sites

Since the reporter gene introduced by AAV vector were driven by the ubiquitous promotor, it is not clear what fraction of somata at either the injection site and/or the terminals at the projection sites are dopaminergic. To estimate the portion of midbrain DA neurons that simultaneously contained the reporter gene of the infected vector (i.e., DsRed2 or tdTomato), we performed double fluorescent IHC against both TH and RFP. We then merged the images to show the co-expression within the somata (Lewis et al. 1988; Blanchard et al. 1994). Figure 1G–I shows representative images of double-labeled neurons within the VTA of M1. Of the RFP^+^ cells around the injection site in M1, 76% (225/291) were TH^+^. In M2, this number was slightly higher, with 79% (206/262) double-labeled neurons. In M3, a smaller number of RFP^+^ cells 37% (100/270) showed concurrent staining for TH. Overall, these results suggest that a considerable portion of the cells expressing RFP were dopaminergic.

### Cortical and Subcortical Projections

**Figure 2:**
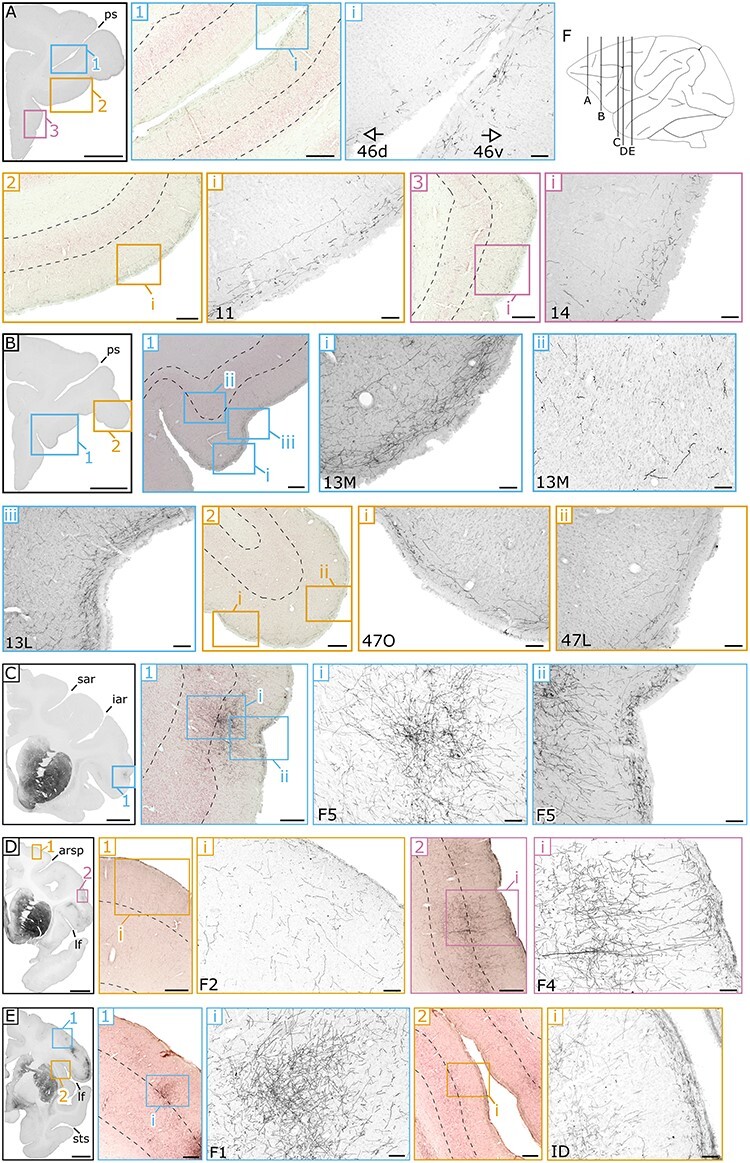
**Example sections of anterograde spread of the viral vector in frontal and somatomotor cortex in M1. (A)** Coronal section at low magnification within the frontal cortex. Within this section, medium-magnification images **(1–3)** show DAB staining of the terminals on top of counterstaining. The dotted lines represent the boundary of the intermediate (laminae II and III) and deeper layers (laminae IV–VI), and that of the deeper layers and the transition into white matter. Images at the highest resolution (roman numerals: **i–iii**) show DAB staining of the axonal fibers in more detail within PFC areas: 46d, 46v, 11 and 14. **(B)** Coronal section posterior to (**A**), with magnified images of PFC areas: 13 M/L and 47 L/O. **(C)** Coronal section highlighting a patch of fibers in motor area F5. **(D)** Coronal section containing 2 additional motor areas of F2 and F4. **(E)** Most posterior coronal section illustrating projections in primary motor area F1 and the dysgranular insula (ID). **(F)** A schematic sagittal view indicating the anterior–posterior position of each of the coronal sections (**A–E**). Scale bar: 5000 μm for **A**, **B**, **C**, **D**, and **E,** 500 μm for **A1, A2, A3, B1, B2**, **C1, D1, D2, E1,** and **E2** and 100 μm for all the other panels. **Landmark sulci**: principal sulcus (ps), superior arcuate sulcus (sar), inferior arcuate sulcus (iar), arcuate rectus spur (arsp), lateral fissure (lf), superior temporal sulcus (sts).

**Figure 3:**
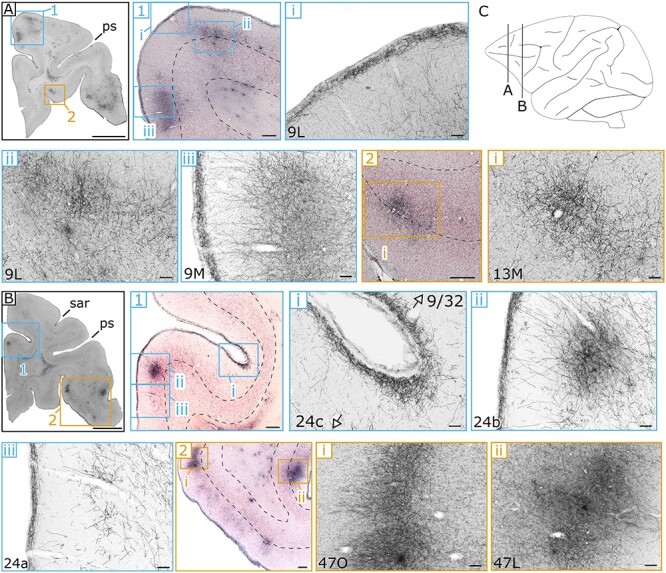
**Example sections of the anterograde spread of viral vector in PFC and the anterior cingulate regions in M3.** Conventions as in Figure 2. **(A)** DAB staining of an anterior coronal section, in which innervations within (1) divisions of area 9 within the DPFC are shown (9 M/9 L) together with (2) a dense patch in the intermediate and deeper layers of OFC area 13 (13 M). **(B)** Coronal section highlighting innervations in (1) the ACC (area 24a-c) and in the bordering DPFC area 9/32. In (2) the more rostral-lateral area 47 (47O/L) of the VLPFC contains concentrated patches mostly restricted to the intermediate layers. **(C)** A schematic sagittal view indicating the anterior–posterior position of each of the coronal sections (A-B). Scale bar: 5000 μm for **A** and **B**, 500 μm for **A1, A2, B1,** and **B2**, and 100 μm for all the other panels. **Landmark sulci**: principal sulcus (ps), superior arcuate sulcus (sar).

**Figure 4:**
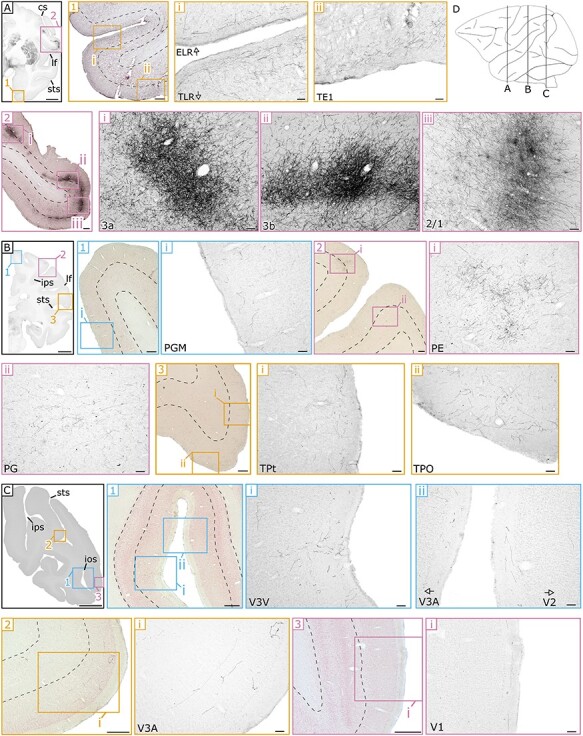
**Example sections of anterograde spread of the viral vector in somatosensory, parieto-occipital, and temporal regions in M1.** Conventions as in Figure 2. **(A)** anterior section showing fiber densities within (1) temporal regions including part of the entorhinal cortex (ELR) and inferior temporal regions (TLR and TE1) and (2) parietal regions including somatosensory areas (3a, 3b, and 2/1) showing dense patches in the intermediate and deeper layers. **(B)** More posterior section highlighting varying fiber densities in (1) dorso-medial parietal area (PGM), (2) intermediate and deeper layers of 2 parietal areas (PE and PG), and (3) in the molecular and intermediate layers of 2 temporal regions (TPt and TPO). **(C)** The most posterior section showing few fibers within the (1–2) intermediate visual regions (V3v, V3A and V2) and (3) hardly any fibers in V1. **(D)** A schematic sagittal view indicating the anterior–posterior position of each of the coronal sections (A-C). Scale bar: 5000 μm for **A, B,** and **C**, 500 μm for **A1, A2, B1, B2, B3, C1, C2,** and **C3**, and 100 μm for all the other panels. **Landmark sulci**: central sulcus (cs), lateral fissure (lf), superior temporal sulcus (sts), intraparietal sulcus (ips), inferior occipital sulcus (ios).

**Figure 5:**
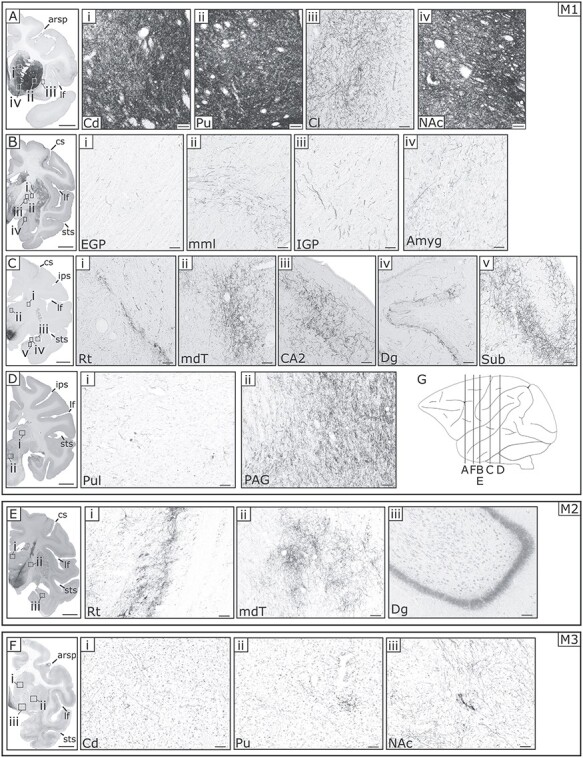
**Example sections highlighting the viral vector spread in a number of selected subcortical regions in M1–3. (A)** Coronal sections of M1 with magnified images (indicated with roman numerals). Significant innervations in the dorsal striatum: (i) caudate nucleus (Cd) and (ii) putamen (Pu). Moderate stained fibers can be seen in the (iii) claustrum (Cl) and in the ventral striatum (iv) nucleus accumbens (NAc). **(B)** Coronal section of M1 with magnified images showing very weak innervations in (i) external globus pallidus (EGP), (ii) medial medullary lamina (mml) a border between 2 basal ganglia nuclei, (iii) internal globus pallidus (IGP), and sparse fiber density in (iv) amygdala (Amyg). **(C)** Coronal section of M1 showing sparse innervations in parts of thalamus and hippocampus: (i) thalamic reticular nucleus (Rt), (ii) medial-dorsal thalamus (mdT), (iii) subfield CA2 of the hippocampus, (iv) the dentate gyrus (Dg), and (v) the subiculum (Sub). **(D)** Coronal section of M1 showing magnified images of the (i) pulvinar (Pul) and (ii) the periaqueductal gray (PAG). **(E)** Coronal section in M2 showing sparse innervations in parts of the thalamus: (i) Rt and (ii) mdT containing a comparable innervation pattern as that in M1, but altogether lacking stained fibers in the hippocampus (iii). **(F)** Coronal section in M3 showing weak and sparse innervations in the dorsal: (i) Cd, (ii) Pu and ventral striatum: (iii) NAc. **(G)** A schematic sagittal view indicating the anterior–posterior position of each of the coronal sections (A–F). Scale bar: 5000 μm for **A, B, C, D, E,** and **F**, and 100 μm for all the other panels. **Landmark sulci**: arcuate rectus spur (arsp), lateral fissure (lf), central sulcus (cs), superior temporal sulcus (sts), intraparietal sulcus (ips).

**Figure 6:**
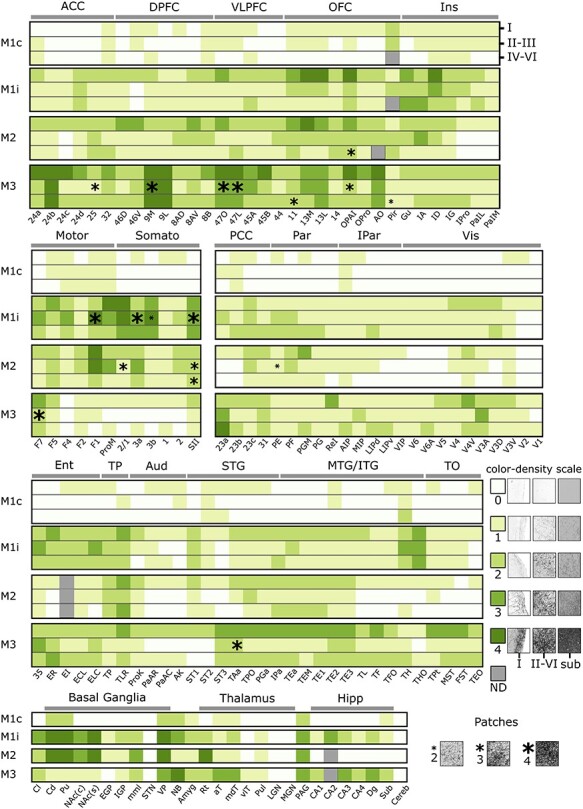
**Heatmap with estimates of fiber densities (ranking) for the areas and laminae throughout the brain of subjects M1-M3.** Rankings for each of the 3 subjects: M1, M2, and M3 with separate rankings in M1 split for contralateral (M1c) and ipsilateral (M1i) hemispheres relative to the injection. The 3 layer segments are separated per row: molecular (lamina I), intermediate (laminae II and III), and deeper layers (laminae IV–VI), as indicated for the first subject (M1c: top right). The scale for the rankings (0–4) is displayed in a legend on the right of the figure, with representative magnified photomicrograph examples of fiber densities at each of the rankings in the molecular layer (I), intermediate and deeper layers combined (II–VI), and for subcortical structures (Sub). In addition, representative examples of patchiness at different strengths (2–4), as conveyed by the size of the asterisk, are shown in the bottom of the figure. The overview consists of 5 major subdivisions, from top to bottom: prefrontal (ACC: anterior cingulate cortex, DPFC: dorsal prefrontal cortex, VLPFC: ventro-lateral prefrontal cortex, OFC: orbitofrontal cortex, Ins: (para)-insular regions), Somatomotor (Motor: motor cortex, Somato: somatosensory cortex), parieto-occipital (PCC: posterior cingulate cortex, Par: parietal cortex, IPar: intraparietal cortex, Vis: visual cortices), temporal (Ent: entorhinal cortex, TP: temporal pole, Aud: (para)-auditory cortex, STG: superior temporal gyrus, MTG/ITG: medial- and inferior-temporal gyri, TO: temporo-occipital cortex) and subcortical (Basal Ganglia, Thalamus and Hipp: hippocampus). ND = nodata.

**Figure 7:**
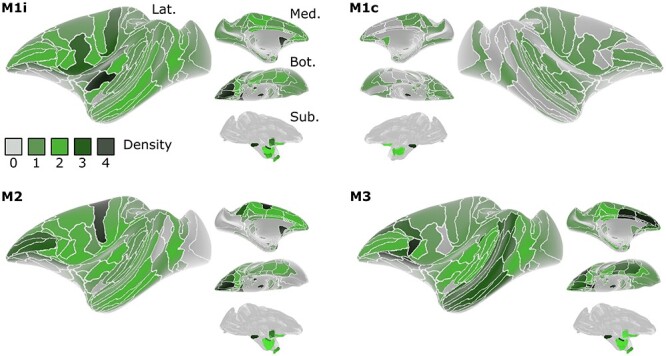
**Schematic overview of fiber densities on the inflated brains of M1-M3, based on the rankings in** Figure 6 **.** The density of estimated terminals (0–4) in the molecular layer of each region is overlaid on a lateral (Lat.), medial (Med.), bottom (Bot.) and subcortical (Sub.) perspective of the inflated D99 brain (Reveley et al. 2017). For M1, both the ipsilateral (M1i) and contralateral (M1c) hemisphere relative to the injection side are shown.

A slice-by-slice assessment of the density of labeled fibers visualized by DAB (see Materials & Methods) revealed how cortical and subcortical structures are innervated by anterograde projections from the ventral midbrain. Stained axons in the cerebral cortex could be broadly divided into 3 categories: those in the most superficial or molecular layer (lamina I), in intermediate layers (laminae II and III), or in the deeper layers (laminae IV–VI). **Figures 2** and **4** collectively highlight example slices at 3 different magnifications of cortical projections in various areas in M1. In Figure 3 representative slices highlighting cortical projections in M3 are presented. Subcortical innervations are indicated in Figure 5, focusing on the most salient results from the 3 animals. In general, the cortical projections found in the molecular layer tended to be more homogeneous compared to VTA projections in intermediate and deeper layers, which sometimes formed concentrated clusters of stained axons that we refer to as patches. The complete overview of cortical projections in the hemisphere ipsilateral to the injection side in subjects M1, M2 and M3 are presented in Supplementary Material S3–S5, respectively. Contralateral projections relative to the injection site were only analyzed in M1 (photomicrographs are not shown in the main text because the stained axons were too sparse). However, see Supplementary Material S6 for a brain-wide overview of the stained coronal sections. The expression pattern of ventral midbrain projections across the entire brain was quantified on a scale from 0 to 4 (visualized in **Figs 6** and **7**).

### Prefrontal Cortex

In light of the previously determined connectivity between the PFC and the midbrain (Williams and Goldman-Rakic 1998), together with a well-established role for DA in cognition within frontal areas (Noudoost and Moore 2011; Ott and Nieder 2019), we first set out to investigate the presence of axonal staining in various regions of the PFC. To this end, we divided frontal cortex into 5 subdivisions, which in turn all contained subregions themselves (overview in Fig. 6). Descriptions of the results below largely follow the rankings presented in Figure 6.

The anterior part of the cingulate (ACC: areas 24, 25, and 32) contains a substantial number of stained axons fibers in the molecular layer for M1 and M2, but received only relatively weak midbrain projections in intermediate and deeper layers. This is in stark contrast with the findings in M3, in which the molecular layer of ACC contains very dense innervations (rank 4: 24a-c, rank 3: 24d, 32, Fig. 3**B1**). In addition, in M3 the cingulate is scattered with strongly stained patches that span mostly the intermediate layers (laminae II and III: Fig. 3**B1ii**), which occasionally cross over into the deeper layers (laminae IV–VI).

The bulk of the PFC encompasses the dorsal prefrontal cortex (DPFC) and ventro-lateral prefrontal cortex (VLPFC). Compared to the ACC, the molecular layer of DPFC of M1 and M2 receives moderate projections from the midbrain (rank 1–2: areas 9 M/L & 8AD, 8B), with an attenuated density in the intermediate and deeper layers. Slightly denser connected regions within the DPFC for these 2 monkeys are area 46 (rank 3 in M2, rank 2 in M1) and the ventral part of area 8A (rank 3 in M2, rank 2 in M1). Within area 46, the density of the terminals seems to increase toward the fundus of the sulcus within the molecular layer (Fig. 2**A1**). What immediately stands out for M3’s DPFC compared to the other subjects is the strong staining across all layers within area 9 (rank 4: 9 M/9 L). Not only does the more homogenous molecular layer color dark, deeply stained patches can be found throughout this area (Fig. 3**A1**). The pattern in the remaining dorsal prefrontal areas in M3 are comparable to those in the other animals, with the notable exception of the deepest layer which ranks moderately, but consistently higher in this animal (rank 0–1: M1 & M2; rank 2–3: M3). The results within the third subdivision of the PFC, that of the VLPFC, do not deviate much from the more dorsal findings above. In M1 and M2, the same superficial to deeper gradient is present, with a medium density in the molecular layer (rank 2: areas 45 & 44) and decreasing innervations in the intermediate and deeper layers (rank 1: areas 45 & 44). Within this subdivision, area 47 is more innervated in M1 and M2 compared to neighboring regions, in particular the orbital part (rank 3: 47O, Fig. 2**B2**). Again, overall staining in M3 was stronger in the VLPFC subdivision, which was most evident in area 45 (rank 3: 45A, rank 4: 45B), but interestingly also in area 47 (rank 3–4: 47O/47L, Fig. 3**B2**).

To glean more insights in the latter observations, we assessed the projections within the more ventral PFC subdivision of the OFC. Although the influence of DA within this frontal part of the brain has been less established than in its more dorsal counterparts (DPFC/VLPFC (Williams and Goldman-Rakic 1998)), deficits in DA to this structure affect various cognitive functions in several animal models (Walker et al. 2009; Zeeb et al. 2010). In all monkeys, overall innervations were stronger in the OFC compared to the subdivisions in the PFC as discussed above. In M1 and M2, the molecular layer was well innervated, which was particularly pronounced in area 11 (rank 3: in both M1 and M2; Fig. 2**A2**), area 13 (rank 4: 13M in M1-M2 and 13L in M1, rank 3: 13L; Fig. 2**B1**), but much less than PFC, and to a lesser extent in area 14 (rank 2 in M1/M2; Fig. 2**A3**). In all of these areas, the axonal densities decrease in a typically cascading fashion toward the intermediate and deeper layers (rank 2: laminae II and III; rank 1: laminae IV–VI). The innervation pattern in M3 resembled that of the other monkeys, with the exception that innervations are typically even stronger, and there are more patchy expressions in the intermediate and deeper layers of area 13 (rank 2–3: 13M and 13L, Fig. 3**A2**). Moreover, small patches were also present in the deepest layers of area 11 in M3. The more posterior OFC regions border the subcortex and consist of the orbital periallocortex (OPAI), orbital proisocortex (OPro), anterior olfactory cortex (AO) and piriform cortex (Pir). Axonal terminal staining in OPAI and OPro was quite similar for all monkeys, with patchy expression in M2 (OPAI: laminae IV–VI) and in M3 (OPAI: laminae II and III). Projections within the anterior olfactory cortex, however, were divergent with relatively few axons in all layers of M1 and M2 (rank 1–2: AO), but significant innervation in M3 (rank 3–4: AO). The piriform cortex is homogenously innervated, with small patches in the deeper layers in M3 (Pir: laminae IV–VI). Interestingly, this structure receives midbrain inputs that are relatively symmetrical across hemispheres (rank 2: Pir of M1c), as opposed to all other frontal structures that we assessed.

The last subdivision within the PFC contains insular and parainsular regions that are roughly located around the inlet of the lateral sulcus. In M1 and M2, axonal densities within this subdivision are unevenly divided. For example, very strong staining is observed in the dorsal insula of M1 (rank 4: ID, Fig. 2 E2), while moderate expression is found in the remaining part of the insula (rank 1–3: IG, IA and gustatory cortex of M1 and M2). Parainsular regions show only weak or even no expression in (rank 0: IPro, PaIL & PaIM in laminae II–VI of M2). In M3, on the other hand, expression is more homogenous throughout the insula, with slightly stronger innervations in the molecular layer compared to the intermediate and deeper layers. In general, all subdivisions within PFC showed a typical trend, receiving dense and diffuse projections in the molecular layer, with the remaining layers receiving patchy projections, which was more pronounced in the intermediate layers.

### Somatomotor Cortex

At first sight, we noticed that expression within the somatosensory and motor cortical areas were quite dissimilar across monkeys. We therefore, decided to consider these areas as a single subdivision to facilitate comparisons in expression patterns. In the more anterior motor regions, the homogeneity of the molecular layer was less pronounced compared to the frontal areas. Rather, the motor and sensory regions consist of dark patches, which are especially prominent in M1 an M2, but mostly absent inM3.

The anterior motor region F7 shows moderate expression in all 3 subjects on average, with a varying distribution of axonal densities in the different layers across the 3 animals. For M3, F7 is the only motor region that is relatively strongly innervated (rank 3: in the molecular layer and patchy expression in the intermediate layers), with the remaining part of the somatomotor cortex showing very weak projections (mostly rank 1, except for F5 and SII with rank 2). The patterns in M1 and M2 are completely different compared to M3, with strong ventral midbrain innervations in primary motor cortex (rank 4: F1, lamina I & laminae II and II in M2; patchy expression in laminae II and III of M1, Fig. 2**E1**). The secondary motor areas also receive stronger projections in M1 and M2 compared to M3. Albeit, less obvious than in F1, these areas also contained dense axonal patches mostly restricted to the intermediate layers (rank 2–3: F5 in M1 and M2, Fig. 2**C1**; rank 1–3: F4 in M1 and M2, Fig. 2**D2i** & rank 1–2: F2 in M1 and M2, Fig. 2**D1i**). Besides the secondary motor areas, strong innervations were present in the more ventral premotor cortex of area ProM, which borders the somatosensory areas (rank 3–4: ProM in M1 and rank 2–3: ProM in M2). As stated above, axonal staining within somatosensory areas of M3 was mostly absent (rank 0–1: 2/1, 3a-b, 1, 2). In contrast, and especially pronounced in M1 but less so in M2, extensive patches were found in the intermediate and deeper layers of areas 2/1 (rank 4: 2/1 in M1, Fig. 4**A2iii**), area 3a (rank 2–3: 3a in M1, Fig. 4**A2i**), and area 3b (rank 3–4: 3b in M1, Fig. 4**A2ii**). The more posterior primary somatosensory areas 1 and 2 contained less axonal terminals and were more equally innervated in the 3 animals (rank 1–2 in M1 and M2; rank 0–1 in M3). The ventrally located secondary somatosensory area showed quite extensive innervations, in particular in the deeper and intermediate layers of M1 (rank 3: SII in all layers, patchiness in laminae II and III) and M2 (rank 2: SII in all layers, patchiness in laminae II–VI).

### Occipito-parietal Cortex

Following the assessment of the frontal PFC subdivisions and somatosensory cortex, we extended our evaluation to the more posterior occipito-parietal areas, which, in light of the classically described anterior to posterior decrease in DA receptor distributions (Berger *et al.* 1988; Gaspar *et al.* 1989), are of particular interest. In agreement with these earlier assessments, the overall axonal densities within these more posterior regions were indeed less pronounced in all 3 animals, most clearly in M1 andM2.

The rankings within the posterior cingulate (PCC) differed only slightly from those described above for ACC. In M1 and M2, diffuse projections targeted area 23, even including some parts not containing any stained axon (rank 1–2: 23a-c in M1; rank 0–2: area 23a-c in M2). A similar situation also held for the more posterior area 31 (rank 1–2: 31 in M1 and M2). Overall axonal densities in M3’s PCC were stronger compared to that of other subjects, exactly as observed in ACC. This was most evident in area 23a (rank 3–4: 23a in laminae II–VI).

The parietal and intraparietal subdivisions occupy the area between the posterior end of somatomotor cortex and the anterior boundary of dorsal extrastriate visual areas. Expression within the inferior parietal lobe areas was sparse and rather similar across monkeys. In M3, only sparse clusters of fibers could be detected within these parietal areas (rank 1–2: PE, PF, PGM, and PG) that were equally divided across the laminae. In M1 and M2 more patchy and dense staining was occasionally observed, for example, in area PE (rank 2: laminae II–VI M1; Fig. 4**B2i**, patchiness in laminae II and III for M2), area PGM (rank 2: laminae IV–VI in M1; Fig. 4**B1i**, rank 3: lamina I in M2) and area PG (rank 1–2: laminae II–VI in M1; Fig. 4**B2ii**). Within the retroinsular region, the pattern was reversed with strong projections in the molecular layer of M3 (rank 3: ReI in M3), but weak innervations throughout all layers of the other 2 monkeys (rank 1: ReI in M1 and M2). Finally, the areas within the intraparietal sulcus receive relatively few midbrain axons. Most pronounced expression is observed in M3, followed by that of M1, and subsequently M2. Overall, staining in M3 is homogeneous but sparse, with small clusters of fibers in the anterior intraparietal area (rank 2: AIP, laminae IV–VI), the medial intraparietal area (rank 2: MIP, laminae II and III) and the ventral and dorsal part of the lateral intraparietal area (rank 2: LIPv & LIPd, laminae IV–VI). Such small axonal clusters were also present in the dorsal part of the lateral intraparietal area of M1 (rank 2: LIPd, laminae IV–VI), although innervations were generally sparse in this intraparietal subdivision. In M2, large portions of the intraparietal subdivision did not contain any labeled fibers (rank 0–1: AIP, MIP, LIPd, LIPv,VIP).

Occipital visual regions were collectively analyzed as a last subdivision within the combined occipito-parietal cortex. Somewhat surprisingly, the general pattern of expression within dorsal visual areas can be seen as an inverted U-shape, in which the more anterior areas V5 and V6 together with the most posterior occipital areas V1 and V2 contain relatively little fibers, at least compared to the more intermediate visual areas V3 and V4. This pattern is most extreme in M2 in which overall expression is low and areas V1/V2 and areas V5 and V6 were completely void of axonal staining (rank 0: V5, V6, V1, V2). In M1, more projections are visible, but most of the anterior and posterior visual regions are weakly innervated by the ventral midbrain (rank 0–1; V5, V6, V1—Fig. 4**C3i**, V2—Fig. 4**C1ii**). In both M1 and M2, the more intermediate visual regions show stronger staining, which is most pronounced in the molecular layer (rank 1–2: V4, V4V, V3A—Fig. 4**C1ii** and **4 C2i**, V3D and V3V—Fig. 4**C1i**) although this extends to the deeper and intermediate layers in some cases (rank 2: V3D in laminae IV–VI of M1). Results in M3 deviate slightly from the other 2 animals. The number of fibers is higher throughout visual cortex, which is most obvious in the intermediate areas (rank 2–3: throughout all laminae in V4V and V3A), but also in the most posterior regions in which projections stretch toward the most posterior end of the occipitallobe.

### Temporal Cortex

Because previous evidence suggests that especially temporal regions of rodents and monkeys differ in terms of DA receptor and terminal distributions (Berger et al. 1991), we set out to investigate projection patterns within several subdivisions along the temporal lobe, roughly from anterior to posterior. Of note is the strong innervation throughout the temporal lobe in the molecular layer of M3, together with local patchy clusters in the temporal pole (TP) and entorhinal cortex. Moreover, cortex is intriguing, the absence of fibers in primary as well as extended auditory cortex.

Expression within the entorhinal cortex was similar in M1 and M3, with a substantial number of fibers throughout area 35 (rank 2: laminae I & IV–VI and rank 3: laminae II and III) and other subregions, including the rostral (rank 2–3: ER, M1 and M3), intermediate (rank 1–2: in EI across layers), caudo-lateral (rank 1–2: ECL throughout all layers), and limiting caudal division of entorhinal cortex (rank 2–3: ELC, M1 and M3). Unfortunately, parts of entorhinal cortex in M2, which mostly included the intermediate zone (EI), were lost during histological preparations. Nonetheless, overall staining in the remaining parts of entorhinal cortex was weak with a minor presence or sometimes absence of axonal terminals in area 35 (rank 1: area 35 in all layers) and the rostral (rank 1: ER, molecular layer, rank 0: laminae II–VI), caudo-lateral (rank 1: ECL in all layers), and limiting caudal division (rank 1: laminae I–III and rank 0: laminae IV–VI) of the entorhinal cortex.

The strongest consistent innervations across the 3 monkeys were found in the areas of the temporal pole. The molecular layer of area TLR contained relatively dense projections in all 3 animals (rank 3: TLR in lamina I, Fig. 4**A1i**). These projections were more variable across monkeys within the deeper layers (rank 1–3: TLR in laminae II–VI, Fig. 4**A1i**). The remaining part of the temporal pole (collectively called TP) receives consistent, but less dense projections in the 3 monkeys (rank 2: all layers for M1 and M2, lamina I in M3; rank 1: laminae II–VI inM3).

Axonal terminals were limited within auditory cortex, in particular in the koniocortex in which the primary auditory cortex itself resides (AK), but also within the belt and parabelt regions (PaAR, PaAC) and in the prosiocortex (ProK). The molecular layer of auditory cortex in M3 was a notable exception, as it showed average staining (rank 2: ProK, PaAR, PaAC & AK). In M1 and M2, the molecular layer was sparsely innervated, and only a handful of fibers was visible in the primary auditory cortex (rank 1: AK), and the rostral (rank 1: PaAR) and caudal (rank 1: PaAC) parabelt regions. The deeper and intermediate layers in all 3 animals were either completely void of midbrain fibers or rarely contained isolated fibers (rank 0–1: ProK/PaAR/PaAC/AK in laminae II–VI).

Various regions along the temporal lobe spanning from the temporal pole to the intraparietal junction and anterior extrastriate visual areas were assessed within the more dorsal superior temporal gyrus (STG), and within the more ventral subdivision of the combined inferior and medial (ITG/MTG) temporal gyrus. Starting with areas within the more dorsal part of the STG: in the anterior regions of ST1 and ST2, expression was restricted to the superficial layer in M3 (rank 2: ST1/ST2). In this animal, the more posterior ST3 showed a substantial upward trend in the number of fibers throughout all layers, but particularly within the molecular layer (rank 3: ST3 lamina I & rank 2: in laminae II–IV). This was a trend in M3, which was also present for the remainder of the areas within the STG located just above the STS (rank 3: TAa/TPO in lamina I; rank 1–2: in laminae II–VI). In M1 and M2, the pattern within the STG was quite similar between the 2 monkeys but deviated from that in M3: in these 2 animals, expression within ST1 and ST2 was sparse but balanced across layers (rank 2: ST1/ST2, lamina I; rank 0–1: laminae II–VI). Within the more posterior superior temporal region of ST3, no expression was observed apart from a couple of fibers in the molecular layer (rank 1: ST3, lamina I). As in M3, more axonal terminals were observed in the ventral regions of the STG bordering the sulcus of these 2 monkeys (rank 2: TAa/TPO, lamina I in M1 and M2; laminae II and III in M1—Fig. 4**B3ii**).

The association areas occupying that part of the STS where the STG transitions into the ITG showed a similar pattern of expression in the 3 monkeys, although overall staining was higher in M3. The more dorsal association area PG, together with the more ventral IPa within the fold of the STS, follows the general trend in which most stained fibers are harbored within the molecular layer (rank 2: PGa/IPa in M1-M2, rank 3: in M3), with more sparse innervations in the intermediate and deeper layers (rank 1–2: PGa/IPa in all 3 animals for laminae II–VI). Largely, the composition of fiber expressions found in the association cortex of the STS was observed throughout most of inferior temporal cortex in both the molecular (rank 3: TEa/TEM/TE1–3 in lamina I for M3; rank 2: in lamina I for M1–2, exemplified by TE1 in Fig. 4**A1ii**) and combined intermediate and deeper layers (rank 1–2: TEa/TEM/TE1–3 in laminae II–VI for M3; rank 0–1: in laminae II–VI for M1–2). Extending into the more MTG, the pattern of expression remained largely the same in M3 (rank 2–3: TL/TF/TFO in lamina I; rank 1–2 in laminae II–VI), while in M1 and M2 the number of fibers decreased. This was most evident in M2 (rank 1–2: TL/TF/TFO in lamina I; rank 0–1 in laminae II–VI). Results from the most medial temporal regions of TH/THO, bordering the entorhinal cortex, were markedly different between the 3 animals. In M1, we discovered dense expressions throughout all layers in these 2 areas (rank 2–3: TH in M1; rank 3: THO in M1). In contrast, in both M3, and especially in M2, the number of fibers was relatively low, if not almost zero (rank 1: TH/THO in lamina I for M2–3; rank 0–2: laminae II–VI).

The posterior temporal areas were grouped into a temporal-occipital (TO) subdivision, located at the confluence between the caudal end of the temporal lobe, parietal cortex and higher order visual areas. The differences between animals are substantial and correspond largely to those described for the visual areas within parietal-occipital cortex. In M3, the molecular layer of these regions receives dense projections (rank 2–3: TPt/MST/FST/TEO, lamina I), which are weaker in the intermediate layers, with small clusters of fibers in the deeper layers (rank 1–2: TPt/MST/FST/TEO, laminae II–VI). In M1, and certainly M2, fibers are mostly absent and if present, they were mostly restricted to the molecular layer (rank 0–1: TPt/MST/FST/TEO in M1 and M2, see Fig. 4**B3i** for an example of areaTPt).

### Subcortical Regions

Alongside an extensive ranking of projections toward the cortex, we also identified several subcortical structures that were of interest in light of potential connectivity with the dopaminergic midbrain. Lacking the typical laminar organization of the cortex, we ranked the density in the individual subcortical regions as a whole. Within the brains of M1 and M2, it was immediately clear that the striatum was preferentially innervated, with extensive staining in both the caudate nucleus (rank 4: Cd—Fig. 5**Ai**) and the putamen (rank 4: Pu—**Fig. 5Aii**). Although less substantial, the dorsal striatal structures in the contralateral hemisphere of M1 also received projections, as evidenced for the putamen (rank 2: Pu) and caudate nucleus (rank 2: Cd). In addition, the ipsilateral ventral striatal nucleus accumbens (NAc) also received considerable projections (Fig. 5**Aiv**), which was slightly more obvious in the shell (rank 4: NAc(s)) compared to the core (rank 3: NAc(c)) of this nucleus. The striatal findings of M1 and M2 are in stark contrast with those of M3, in which only sparse projections could be detected in the caudate nucleus (rank 1: Cd, Fig. 5**Fi**), putamen (rank 1: Pu, Fig. 5**Fii**) and the shell (rank 1: NAc(s)) and core (rank 1: NAc(c)) of the nucleus accumbens (Fig. 5**Fiii**).

The expression profile of the remaining structures of the basal ganglia that we ranked was more comparable across monkeys. The external and internal parts of the globus pallidus show relatively little or even no AAV expression (rank 1: EGP in M1–2, Fig. 5**Bi**, rank 0: M3; rank 2: IGP in M1, rank 1: M2–3, Fig. 5**Biii**), whereas the medial medullary lamina (mml) separating the 2 related nuclei is clearly visible, containing a relatively higher number of fibers (rank 2: mml in M1/3—Fig. 5**Bii**, rank 3: M2). We were unable to find any expression in the subthalamic nucleus, apart from those related to the trajectory of the injections (rank 0: STN). This suggests that the SNc was relatively weakly transduced by our injections, even in M1 and M2. Indeed, at least in rodents, the STN receives dopaminergic input from the latter structure (Cragg et al. 2004). Finally, the ventral pallidum, an important component of the limbic system and part of the basal ganglia, showed dense staining in all monkeys (rank 3–4: VP, M1–3), even within the contralateral hemisphere of M1 (rank 2: VP,M1c).

Three other subcortical regions, which we deemed of interest, are the claustrum (Cl), nucleus basalis (NB), and the amygdala (Amyg). Fiber density within the claustrum was high and rather homogenous from its more anterior to posterior extension in M1 (rank 3: Cl, Fig. 5**Aiii**) and in M2–3 (rank 2: Cl). The cholinergic forebrain region of the nucleus basalis is located ventral to the neighboring globus pallidus and ventral pallidum and received very strong projections in M3 (rank 4: NB), with a smaller, yet symmetrical, distribution of fibers in M1 (rank 3: NB, M1, ipsilateral; rank 2: M1, contralateral) and almost no fibers in M2 (rank 1: NB). Although the amygdala consists of several divisions, we did not distinguish these as subregions and ranked the structure as a whole. In fact, the density of the projections was relatively low in any of the subjects (rank 1–2: Amyg in M1–3).

To gain an in-depth insight into thalamic innervations from the dopaminergic midbrain, we split the structure into 7 of its main components that could be identified based on anatomical landmarks. We found that the reticular nucleus (or pre-thalamus) ranked high in M2 (rank 4: Rt, Fig. 5**Ei**) and a relatively high density of fibers was also present in the other 2 animals (rank 2: Rt, Fig. 5**Ci**). Other, moderately innervated thalamic divisions are the medial-dorsal (mdT) and anterior (aT) part, in which M3 received more projections (rank 3: aT/mdT) than M1 (rank 2: mdT, Fig. 5**Cii**; rank 1: aT) and M2 (rank 1: mdT/aT, Fig. 5**Eii** which shows the mdT). Both the pulvinar (Pul) and the ventro-lateral thalamus (vlT) contained little to no fibers (rank 1: Pul in M1-M3; rank 0: vlT in M1–2 and rank 1: M3). Finally, the 2 primary sensory nuclei at the foot of the thalamus were completely void of midbrain fibers, as evidenced by the absence of projections in both the lateral (rank 0: LGN in M1–3) and medial geniculate nucleus (rank 0: MGN in M1–3).

Axonal fibers in the hippocampus were estimated based on their location within the CA subfields (CA1–4), the dentate gyrus (Dg), and subiculum (Sub). In 2 of the animals, the tiny CA2 subfield was almost impossible to distinguish, but in M1 we noticed that his area preferentially received dense expressions (rank 3: CA2, Fig. 5**Ciii**). The other subfields also contained fiber staining which was higher on average for M3 (rank 3: CA3, rank 2: CA1/4) compared to M1 (rank 2: CA3, rank 1: CA1/4). Similarly, in the remainder of the hippocampal subregions staining for the dentate gyrus and subiculum was higher in M3 (rank 3: Dg, rank 2: Sub) compared to M1 (rank 2: Dg, Fig. 5**Civ**; rank 1: Sub, Fig. 5**Cv**). A profoundly aberrant picture of expressions was observed in subject M2, in which, interestingly, none of the hippocampal subdivisions contained any stained fiber (rank 0: CA1–4, Dg—Fig. 5**Eiii**,Sub).

Two more posteriorly located subcortical regions that we considered separately in our rankings are the midbrain gray matter area of the periaqueductal gray (PAG) enveloped around the fourth ventricle, and the cerebellum (Cereb). Along the PAG, we discovered strong staining that was consistent across monkeys (rank 3: PAG in M1-M3) and even transcended into the contralateral hemisphere in M1 (rank 2: PAG in M1c). We decided not to further split up the cerebellum, since we did not discover a single fiber throughout this large brain structure in any of the animals (rank 0: Cereb M1-M3).

### IHC Double Labeling of Nerve Terminals

**Figure 8:**
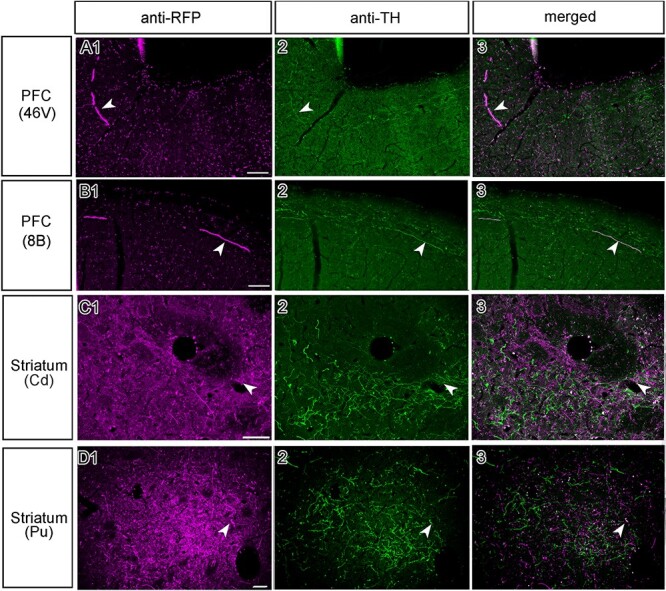
**Double IHC of axonal terminals originating from the midbrain does not indicate clear co-localization in M1. (A)** Selective photomicrographs of the prefrontal cortex (area 46 V), showing staining against (1) RFP, (2) TH, and (3) a merged image, with the white arrowheads marking an example of terminals that are potentially double-labeled. Here, the double-labeling was rare. **(B)** A photomicrograph from area 8B, indicating the potential co-localization with anti-RFP and anti-TH staining, again double-labeling was rare. **(C)** An optical sectioning image of double fluorescent IHC within the caudate nucleus revealed more DAergic (TH^+^) terminals, but not necessarily more co-localized (RFP^+^) fibers, which was also the case in **(D)** for the putamen. Scale bar: 100 μm.

As described above, a substantial portion of VTA neurons transfected with AAV were dopaminergic. It is unclear whether this also applies for the axon terminals in cortical targets. To address this question, we performed IHC double-labeling using anti-RFP and anti-TH antibodies on a handful of coronal slices of M1 (Fig. 8). Within a representative frontal section through DPFC including area 46 V, only a small number of axons were double-labeled for both the reporter gene and TH (Fig. 8A: as indicated with white arrowheads). This also held true for axons in the molecular layer of a subregion within DPFC (i.e., area 8B**,**Fig. 8B). These results seem to imply that a majority of cortical VTA projections are non-dopaminergic. To substantiate such a counterintuitive conclusion, however, we also double-labeled parts of the brain known to contain strong dopaminergic input from the VTA, including the striatum. Surprisingly, even within striatal components, such as the caudate nucleus (Fig. 8C) and the putamen (Fig. 8D), we encountered only a few double-labeled fibers. It is therefore tempting to relate the absence of double-labeling of axons in general, to technical limitations instead, as we will further discuss below.

## Discussion

By employing state-of-the-art anterograde viral tract tracing techniques, this is the first study to provide an overview of the detailed axonal arborization of the VTA throughout the entire brain of macaque. Dopaminergic neuronal projections from the midbrain in nonhuman primates have been studied extensively, but essentially in reverse, i.e., by using more traditional retrograde tracing techniques. By definition, such retrograde studies can only provide a snapshot of the full connectome from any given structure. Nonetheless, these retrograde studies revealed that the dopaminergic midbrain is preferentially connected to a variety of areas within the frontal cortex (Williams and Goldman-Rakic 1998; Frankle *et al.* 2006). Moreover, it was found that these midbrain-frontal projections originated in the 3 major midbrain nuclei housing dopaminergic neurons: i.e., A10 (VTA), A9 (SNc), and A8 (RRF). To provide a more detailed and comprehensive understanding of the brain-wide distribution of the DA terminals, we aimed to target the VTA using our anterograde vectors. Subsequently, we assessed the relative intensity of VTA projections in individual areas and laminae throughout the brain.

In chronological order of the experiments, we first used AAV-DJ in M2 and M3. AAV-DJ induced strong expression of the FP, but seemed to have caused some minor damage to the tissue presumably due to inflammation. Therefore, the third monkey we injected (M1), we chose an alternative serotype, AAV2.1, which did not appear to be inflammatory. In this case, we tried to intensify the signals of anti-FP IHC more to compensate for the weaker expression of FP. In addition, we incorporated ChR2 into the viral vector to examine the expression of ChR2 in the axons originating in the VTA for potential optogenetic stimulation experiments. The present results indicate that integration of ChR2 did not appear to seriously harm the labeling of the somas and axons of VTA neurons.

Previous studies showed that the highest concentrations of cortical catecholaminergic (i.e., dopaminergic and noradrenergic) terminals are found in prefrontal and temporal cortex, with decreasing levels in premotor and occipital cortex (Mac Brown and Goldman 1977; Björklund et al. 1978). A follow-up study showed widespread distribution of TH^+^ fibers, indicative of their dopaminergic or noradrenergic nature, throughout the cerebral cortex of macaques (Lewis et al. 1987). These studies describe the ubiquity of DA innervations in primates arguing against pronounced regionally restricted projections (i.e., within frontal and striatal regions). However, information regarding the exact nature and origin of the catecholaminergic innervation remained obscure. These TH^+^ axons could be dopaminergic projections from either the VTA, SNc, or RRF, or noradrenergic projections from the locus coeruleus, despite morphological differences in nerve terminals were proposed (Levitt et al. 1984). More recently, anatomical features of dopaminergic midbrain nuclei have been investigated in much more detail in rodents, through genetically defined subgroups that innervate divergent brain targets: the more lateral SNc projects to striatal targets (caudate, putamen), whereas the more medial VTA projects more into the nucleus accumbens, olfactory tubercle and the forebrain (Poulin et al. 2018). Whether these findings can be directly compared to the spatial organization of the DA system in primates, however, is questionable, especially since areal and laminar DA receptor distributions differ profoundly between non-human primates and rodents (Berger *et al.* 1991). It is reasonable to assume that the DA system has undergone a significant transformation ever since the evolutionary split of the rodent and primate lines. Such a transformation emphasizes the necessity to investigate the exact DA connectivity profile in terms of both their midbrain origins and subsequent projection sites in primates.

Our aim was to target the VTA (A10). Since this nucleus has an irregular border with the neighboring SNc (A9) and RRF (A8) (Fig. 1), however, it is exceedingly difficult to confine an injection to this tiny region. Based on the histological assessment of the exact injection sites and the spread of transduced neurons, we could basically divide our subjects into 2 groups: (1) M1 and M2, in which the vector transduced neurons in the VTA but also a part of the SNc, and (2) M3, in which expression was present in the more dorsal part of the VTA, in addition to parts of the ventro-medial thalamus but sparing the SNc. It is important to note that in all the animals, the injection avoided theRRF.

In M1 and M2, densest projections were observed in the OFC and sensorimotor regions (with patchy projections in the deeper layers). In PFC of M3, on the other hand, strong projections were found in the OFC, DPFC, VLPFC, and ACC (Fig. 7 for an overview). The consistent presence of dense innervations within OFC of all 3 subjects is the most resolute evidence that this subdivision is most strongly innervated by the VTA, at least compared to the other PFC subdivisions. Within subcortical regions, a strikingly different expression pattern was observed in the striatum, which received strong projections in subjects M1 and M2, but not in M3. Although speculative, these differences could be explained by the fact that the dorsal striatum is mainly targeted by the SNc and the ventral striatum by ventral VTA or medial SNc (Gerfen 1985). A peculiar difference between the first 2 monkeys was observed in the hippocampus, which received weak versus no projections in M1 and M2, respectively. In M1, slightly more medial portions of the VTA are transduced compared to M2 (Fig. 1D and E). Since in M3 the most medially located VTA neurons were transduced (Fig. 1C), and since also the hippocampus in M3 was strongly innervated, it may be that the hippocampus is mainly innervated by the most medial part of the VTA, arguing for a specific functional topography within the VTA (Lisman and Grace 2005).

Despite the intrusion of our vector in the SNc and ventro-medial thalamus in M1/M2 and M3, respectively, our results suggest that projections originating in the VTA are most likely not homogeneous. There appears to be a gradient from medial to lateral VTA, with the lateral part more strongly connected to the somatomotor cortex and the medial part to areas V3 and V4 and the temporal lobe. Previous electrophysiological studies provided corroborating evidence for a heterogeneous VTA/SNc (Matsumoto and Hikosaka 2009). Awaiting confirmation of heterogeneous connectivity patterns originating in the VTA, one can speculate about different functional roles of the medial and more lateral parts of the VTA. For example, the latter may be involved in reinforcement-based motor learning, and the medial part in reinforcement-based visual associative learning. With respect to the latter conjecture, it is interesting to note that, in a completely independent study, we observed cortical plasticity in posterior area PIT and V3 driven by electrical stimulation of the VTA in the presence of a very weak unattended visual stimulus (Arsenault and Vanduffel 2019). In parallel, one of the authors previously showed that the NAc facilitates motor cortical activity and contributes to recovery from spinal cord injury in macaques (Sawada et al. 2015). We presume that the lateral VTA mediates the inputs from the NAc to the motor cortex in thiscase.

The main conclusion that we derive from these results is that VTA-originating projections cover the entire brain, with denser innervation in the more frontal regions and sparse projections in the posterior part of the brain, exactly as suggested in previous studies (Berger *et al.* 1988; Williams and Goldman-Rakic 1998). We suspect that the hotspots of innervations observed in idiosyncratic cases are the result of the disparity of the injections. For example, the predominantly medial VTA injection in M3 might have resulted in particularly dense projections to the OFC and other prefrontal regions. It is tempting to speculate that the slightly more lateral SNc injection in M1-M2 led to the strong innervations in the striatum and sensorimotor areas, which were lacking in M3. Of note, the lack of strong striatal innervations within M3 might suggest that the observed projections within the DPFC, ACC, VLPFC partially originated in the ventro-medial thalamus in addition to the medial VTA (Kolb et al. 1982; McFarland and Haber 2002). The combined results suggest a segregation of midbrain dopaminergic neurons in the lateral VTA and SNc versus medial VTA and ventro-medial thalamus in terms of their cortical and subcortical targets. This anatomical segregation is interesting in light of recent behavioral findings on the functional topography in rodents through photostimulation of the more medial VTA versus that of the more lateral VTA/SNc (Keiflin et al. 2019). Similarly, a broad medio-lateral physiological segregation based on variable electrophysiological outcomes in DA neurons has been proposed previously in macaques (Matsumoto and Hikosaka 2009). The relationship between the observed anatomical and physiological segregation of DA neuron subgroups should be studied in more detail with state-of-the-art selective manipulation techniques (Vancraeyenest *et al.* 2020) in future primate studies.

It is important to note that the injected vector tracers we used in all our subjects are non cell–type-specific. Although viable TH-specific promoters are in circulation and have been successfully used in previous work (Lerchner et al. 2014; Stauffer *et al.* 2016), there are some concerns about the transfection efficiency and specificity of these promoters. Rough estimates indicate that only 40% of the TH^+^ cells are infected by these injected cell-type specific vectors (Lohani et al. 2017; Lohani et al. 2018). We used the generic non-cell type-specific CAG promoter as it drives high levels of long-term gene expression (Alexopoulou et al. 2008). Our choice for a generic non-cell type-specific promoter means that we cannot be conclusive about the dopaminergic nature of the stained axons in the projection sites. However, representative double-labeled fluorescence samples indicate that at least a substantial fraction of the somata within the midbrain (Fig. 1G–I), and some of their axons in projection sites are dopaminergic (Fig. 8**A1–D3**), as they are both RFP^+^ (i.e., transduced by the vector) and TH^+^ (dopaminergic). Previous studies by Goldman-Rakic and colleagues suggested that dopaminergic projections to temporal and frontal lobes exhibit a bimodal distribution, with one peak in more superficial layers (laminae II and III) and a second peak in deeper layers (laminae IV and V) (Levitt *et al.* 1984). These findings were substantiated by rich expression of D1-type dopaminergic receptors, again both in the superficial and deeper layers in different parts of the neocortex, while expression of D2 receptors was mostly restricted to layer V (Lidow *et al.* 1991). A more recent study on the other hand showed a more ubiquitous distribution of D1- and D2-type receptor proteins in the cell bodies of neurons across laminae II–VI in the frontal eye field of macaques (Mueller et al. 2020). To clarify whether the cortical projection fibers in the superficial and deep layers are dopaminergic, we performed double IHC staining in the cortex. However, observations of double-labeled axon terminals were rare (Fig. 8A and B), even when a majority of RFP^+^ cell bodies within the VTA were double-labeled (Fig. 1I**)**. Surprisingly, even in the striatum only a low number of RFP^+^ axons were also stained for TH (Fig. 8C and D). Yet, the majority of striatal axons originating in the ventral midbrain are considered to be dopaminergic, at least in rodents (Ikemoto 2007). How can we explain these seemingly false negative results? Recently, it has been reported that it is challenging to detect anti-TH immuno-staining in neurons transduced by an AAV vector because of possible downregulation of TH synthesis in the infected neurons (Albert et al. 2019). Therefore, the negative evidence in the present study may be related to unknown technical difficulties to show that RFP^+^ axons are TH^+^ after transduction with an AAV vector. In conclusion, our axonal double-labeling data have to be interpreted with caution and should not be taken as conclusive evidence that the RFP^+^ axons are actually non-dopaminergic.

When comparing our anatomical connectivity results with those of our previous “effective connectivity” findings obtained through a combination of electrical stimulation of the VTA (VTA-EM) and fMRI, we found similarities and differences. Major cortical regions showing increased fMRI activity following VTA-EM included frontal areas such as the PFC and OFC, ACC, intraparietal areas, as well as somato-motor regions, insula, and gustatory cortex (Arsenault *et al.* 2014), exactly as observed in the present tracer study, in particular in subjects M1 and M2. In addition, subcortical regions showing VTA-stimulation induced activity included the caudate, putamen, and parts of the thalamus (Arsenault *et al.* 2014). The OFC, parts of prefrontal and temporal cortex, and to some extent more posterior parietal and visual areas were activated after VTA stimulation at intermediate stimulation frequencies (Murris *et al.* 2020), a connectivity map that more closely resembles the findings in M3. In the latter VTA-EM study, activation of somatomotor areas and the striatum was weak, although these areas showed strong midbrain projections in M1 and M2. Hence, it is tempting to relate the differences in vector spread in the present study to the exact positioning of the chronic electrodes in the VTA-EM studies above.

The present study includes an extensive analysis of both cortical and subcortical DA projections revealing interregional and laminar-specific similarities and differences across injected animals. The differences may be attributed to slight differences in the spread of the vector. The main novelty of this VTA-DA anatomical connectivity study is that by using anterograde tracers, we could provide a detailed efferent connectivity map for the DA in correspondence with midbrain of non-human primates (Figs 6 and 7). We observed clear laminar specificity in the projections, with strongest innervation of the molecular layer in many cortical areas, and with sometimes dense patches resembling parts of columnar-like structures in deeper layers of specific brain regions. In light of inter-species (Raghanti et al. 2008) and regional differences in D1/D2 receptor distributions (Lidow *et al.* 1991), these findings will be highly instrumental to guide experiments aimed to study the functional impact of dopaminergic-specific midbrain projections on cortical and sub-cortical processing.

## Notes

The animals (*Macaca fuscata*) were provided by the National BioResource Project-Nihonzaru (Japanese macaque) at Kyoto University Primate Research Institute with support in part by the National BioResource Project of the Japan Agency for Medical Research and Development (NBRP). We thank J. Arsenault for instructing the injection protocol and M. Takada and K. Inoue for providing us the plasmids of AAV2.1 vector. We thank K. Svoboda for providing the gene AAV-CAG-hChR2-H134R-tdTomato. We also thank J. Yamashita, M. Nakamura and Y. Shinto for technical assistances. We thank X. Li and Q. Zhu for their assistance with the renderings in Figure 7. *Conflict of Interest*: The authors declare no conflict of interest.

## Funding

This work was supported by Kyoto University; Japanese KAKENHI Grants from the Ministry of Education, Culture, Sports, Science and Technology of Japan (26112008, 19H05723 and 19KK0192), Japan Society for the Promotion of Science International Bilateral Joint Research Program, the Japan Agency of Science and Technology/Core Researches for Evolutionary Science and Technology (JPMJCR1651), the Japan Agency for Medical Research and Development (20DM0107151 and 20DM0307005); Leuven (C14/17/109); Fonds Wetenschappelijk Onderzoek-Vlaanderen (FWO-Flanders) (G0D5817N, G0B8617N, G0E0520N, VS02219N; and Odysseus G0007.12); and the European Union’s Horizon 2020 Framework Programme for Research and Innovation under Grant Agreement No 945539 (Human Brain Project SGA3).

## Supplementary Material

Supplementary_20201212_bhaa399

## Data Availability

The datasets used in the current study are freely available on the Human Brain Project (https://search.kg.ebrains.eu/instances/Dataset/f6d8e6a8-6f24-48b5-8187-28d8d7e63219). The dataset DOI is: 10.25493/R726-Q8Q.
