# Supplementary material for: Divergent Whole Brain Projections from the Ventral Midbrain in Macaques
Source: Cereb Cortex. 2021 Feb 9;31(6):2913–31. doi: 10.1093/cercor/bhaa399 (PMC8107798; doi:10.1093/cercor/bhaa399)
Supplement: Supplementary_20201212_bhaa399 [file supplementary_20201212_bhaa399.docx]

**Supplementary Material**

**Divergent whole brain projections from the ventral midbrain in macaques**

Muhammad Zubair, Sjoerd R Murris, Kaoru Isa, Hirotaka Onoe, Yoshinori Koshimizu, Kenta Kobayashi, Wim Vanduffel, Tadashi Isa

| **Subject** | **M1** | **M2** | **M3** |
| --- | --- | --- | --- |
| **sex** | Female | Female | Female |
| **age (year)** | 4 | 7 | 9 |
| **body weight (kg)** | 4.2 | 6.7 | 9.4 |
| **vector serotype** | AAV2.1 | AAV DJ | AAV DJ |
| **vector FP-tag** | DsRed2 | tdTomato | tdTomato |
| **vector titer**  **(unit vg/mL)** | 2.2x10^12^ | 1.1x10^13^ | 3.8x10^12^ |
| **injected side** | Left | Left | Left |
| **volume**  **/injection site (μL)** | 1.0 | 0.5 | 0.7 |
| **injection rate (nL/min)** | 100 | 100 | 100 |
| **tracks (#)** | 3 | 3 | 3 |
| **injection sites**  **/tracks (#)** | 1 | 1 | 1-2 |
| **total number of injection sites** | 3 | 3 | 5 |
| **days until perfusion** | 41 | 42 | 42 |

**Supplement 1: Subject and experimental characteristics**

A comprehensive overview: which includes specifications of the animals, the viral vectors and the injection procedures in the study. The first four rows refer to the animals (*Macaca fuscata*) that were used in this study and their weight and age at the time of perfusion. Three rows below provide information on the serotype of the vector, the fluorescent tag that could either directly be used in histology for fluorescence staining or through enhanced staining (DAB), and the predetermined vector titer (see Materials & Methods). The bottom six rows relate to details on the injection procedures that differed only slightly between subjects. In all animals, the left hemisphere was injected, with the main difference being the injected volume, which was more in M3, followed by that in M1 and M2.


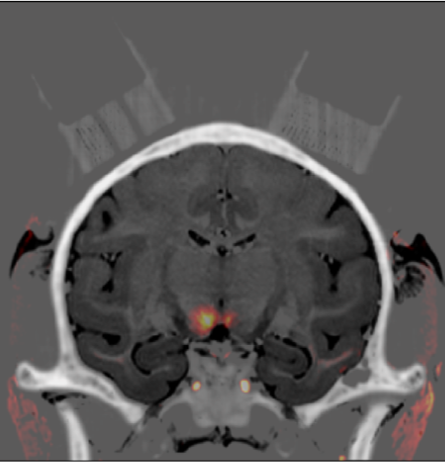


**Supplement 2: Pre-injection targeting of the VTA through a combination of CT and contrast enhanced MR imaging.** The midpoint of a gadolinium injection targeting the right VTA (T1 in hot metal color scale) was overlaid on a representative T2-reverse MRI coronal slice and CT (both in gray scale) showing anatomical outlines, the skull and implanted chamber/grids. Individual pre-operative imaging facilitated in determining the exact injection site in each animal, in addition to providing a reliable estimate of potential vector spread when taking gadolinium as a reference, before moving on to the actual viral vector injections.


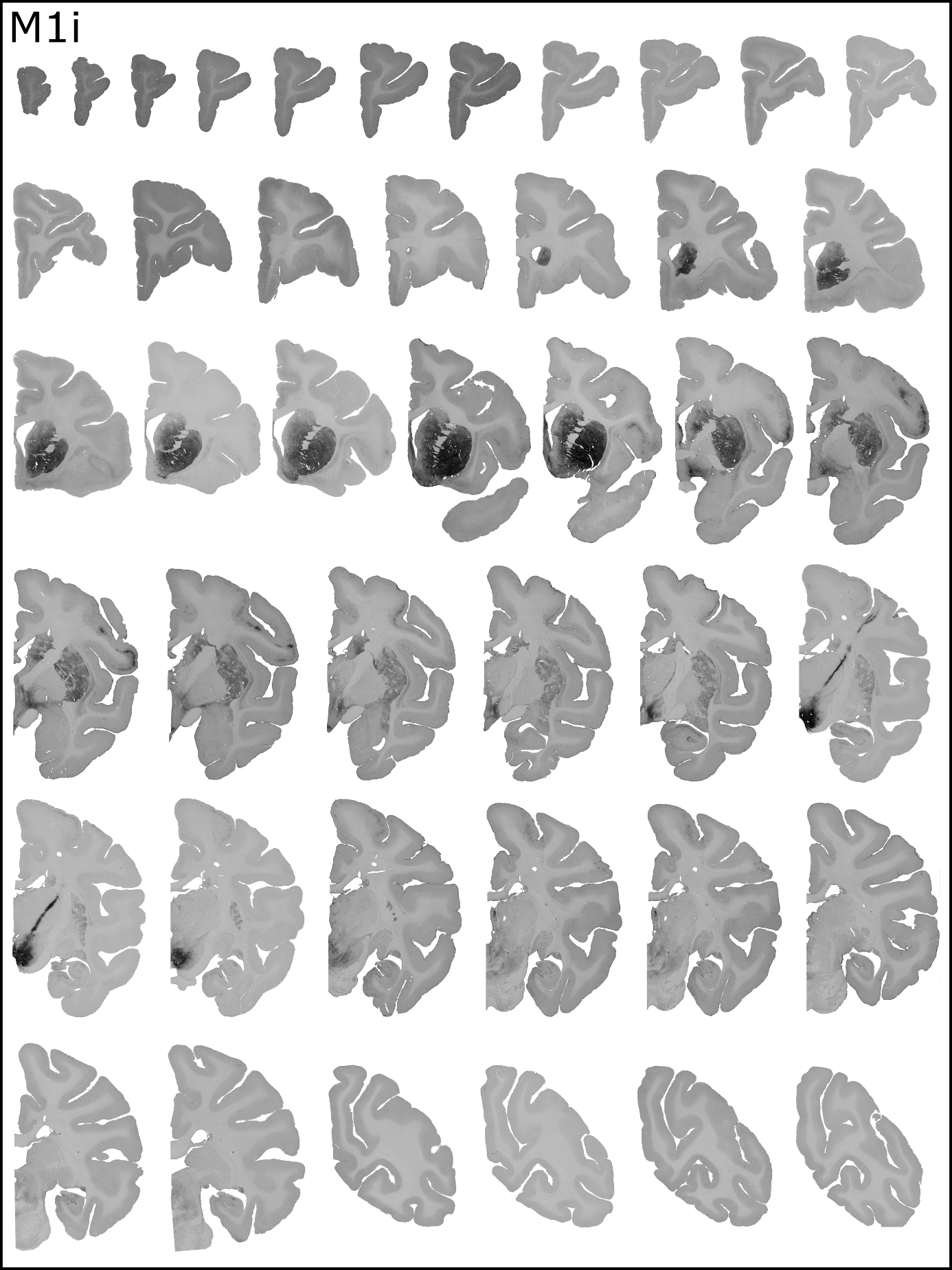


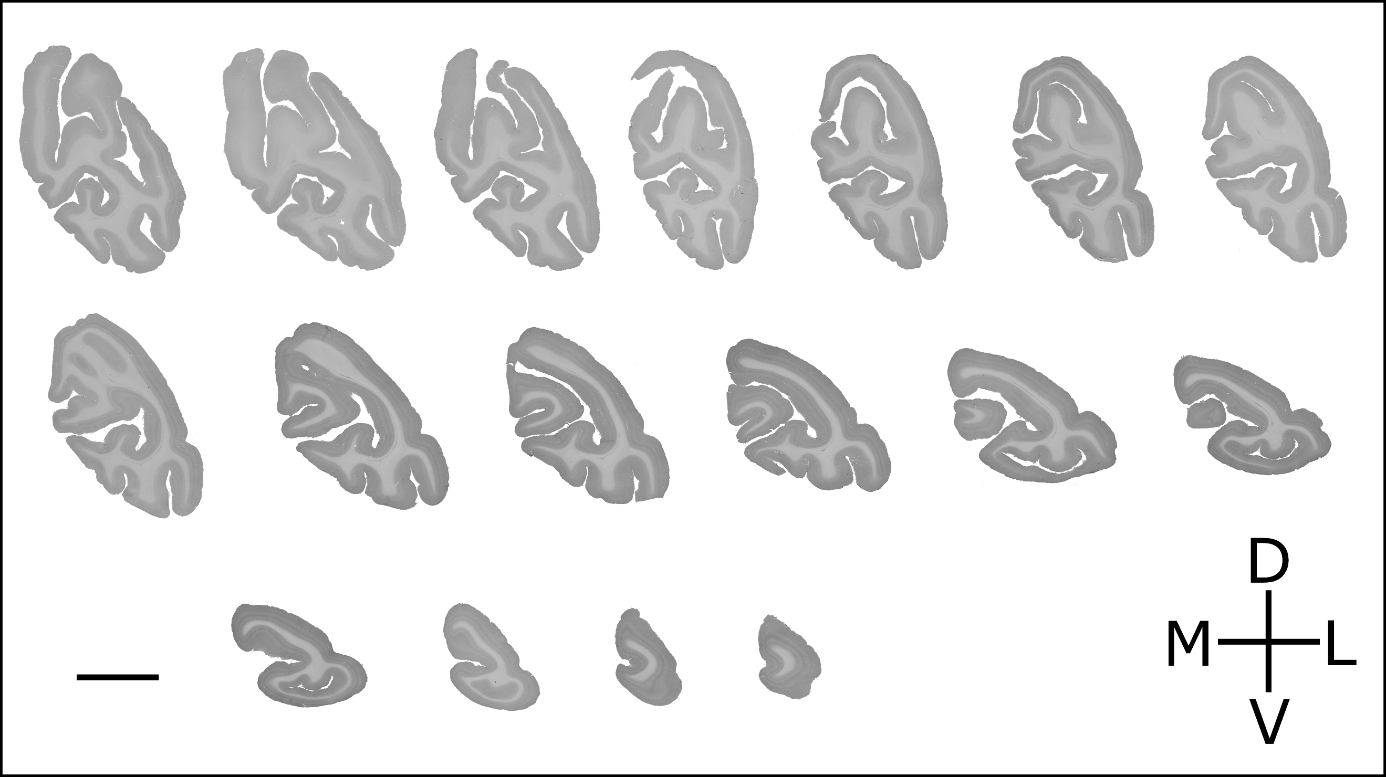


**Supplement 3: Overview of all IHC stained coronal sections of the hemisphere ipsilateral to the injection in M1.** The coronal sections shown here at low magnification were used to determine the fiber density from DAB staining throughout the brain in M1 (see Figure 6-7) and therefore relate to the main findings in this study. The presentation of the coronal slices is from anterior (top left) to posterior (bottom right). Scale bar (bottom left): 10 mm. **Coordinate system**: dorsal (D), lateral (L), ventral (V), medial (M).


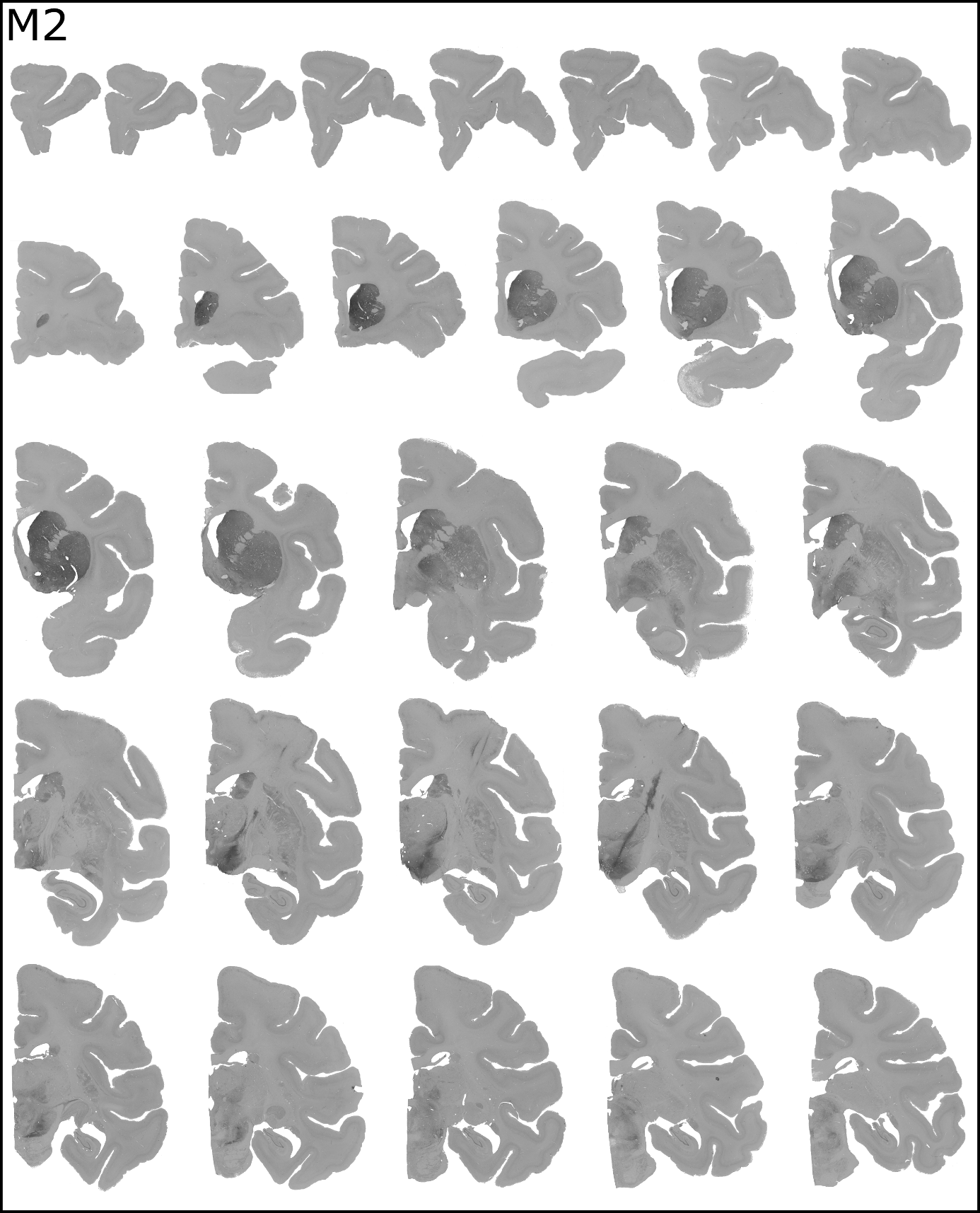


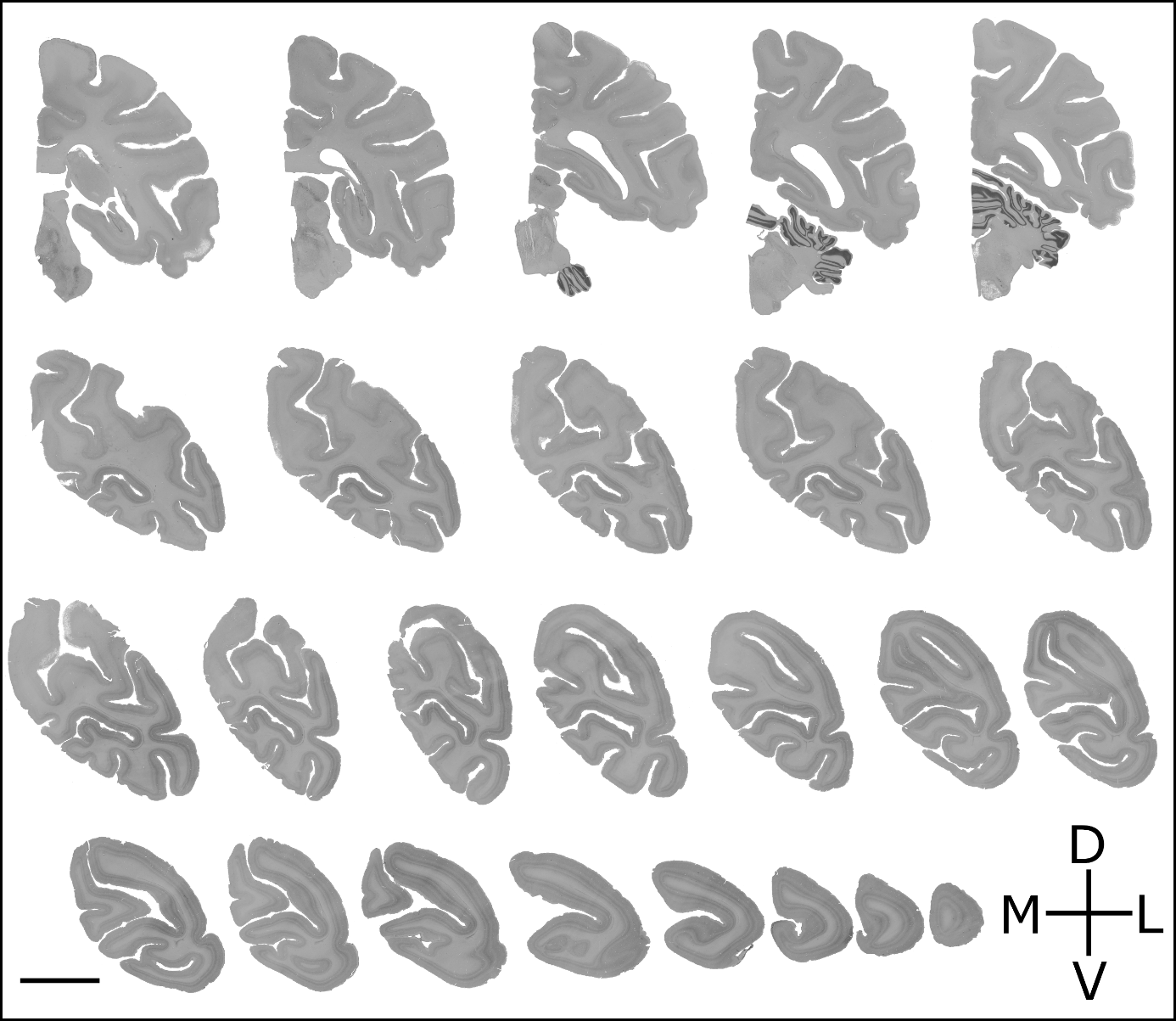


**Supplement 4: Overview of all IHC stained coronal sections of the hemisphere ipsilateral to the injection in M2.** Conventions follow those in Supplement 3; but presented coronal slices are from the ipsilateral hemisphere in M2.


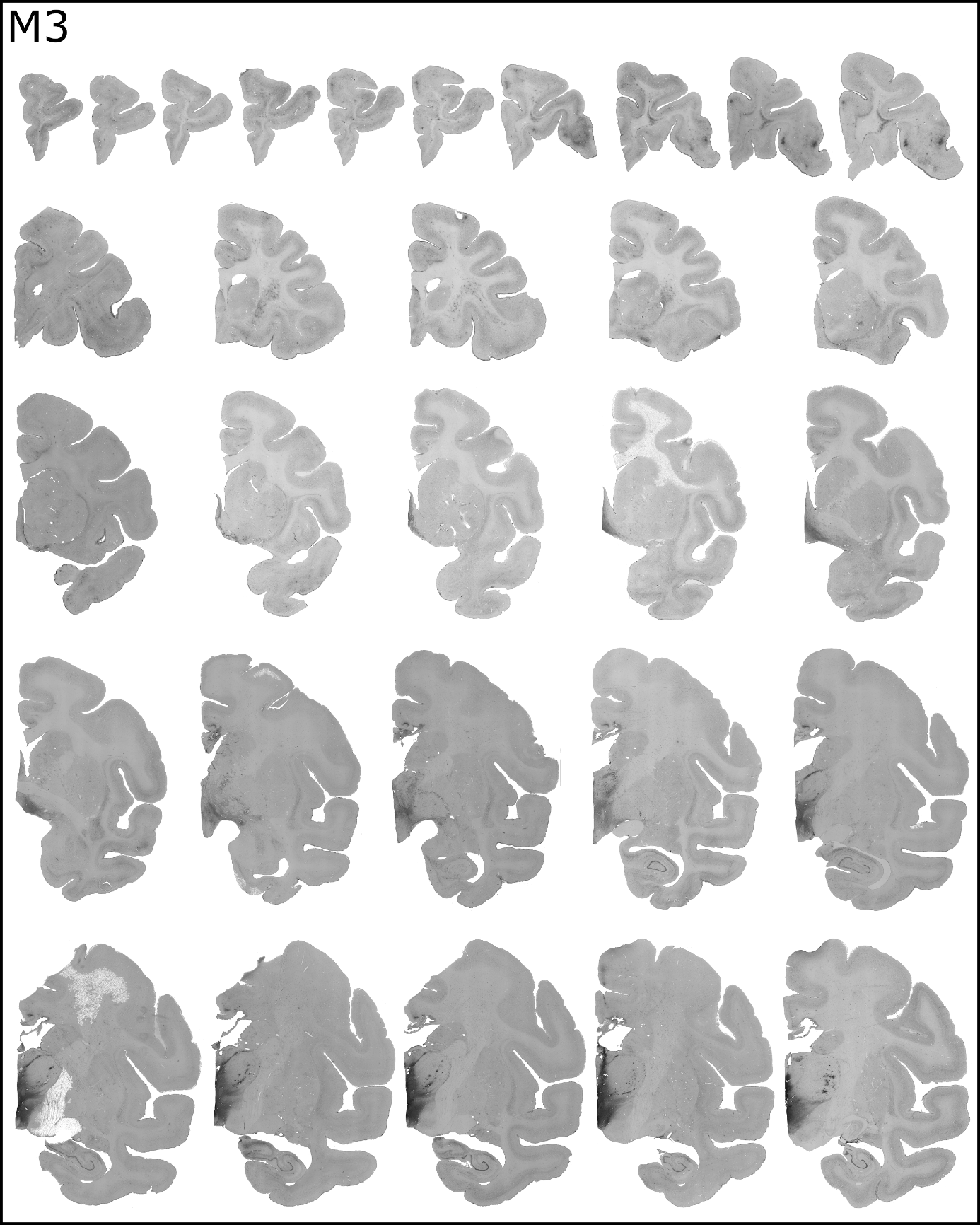


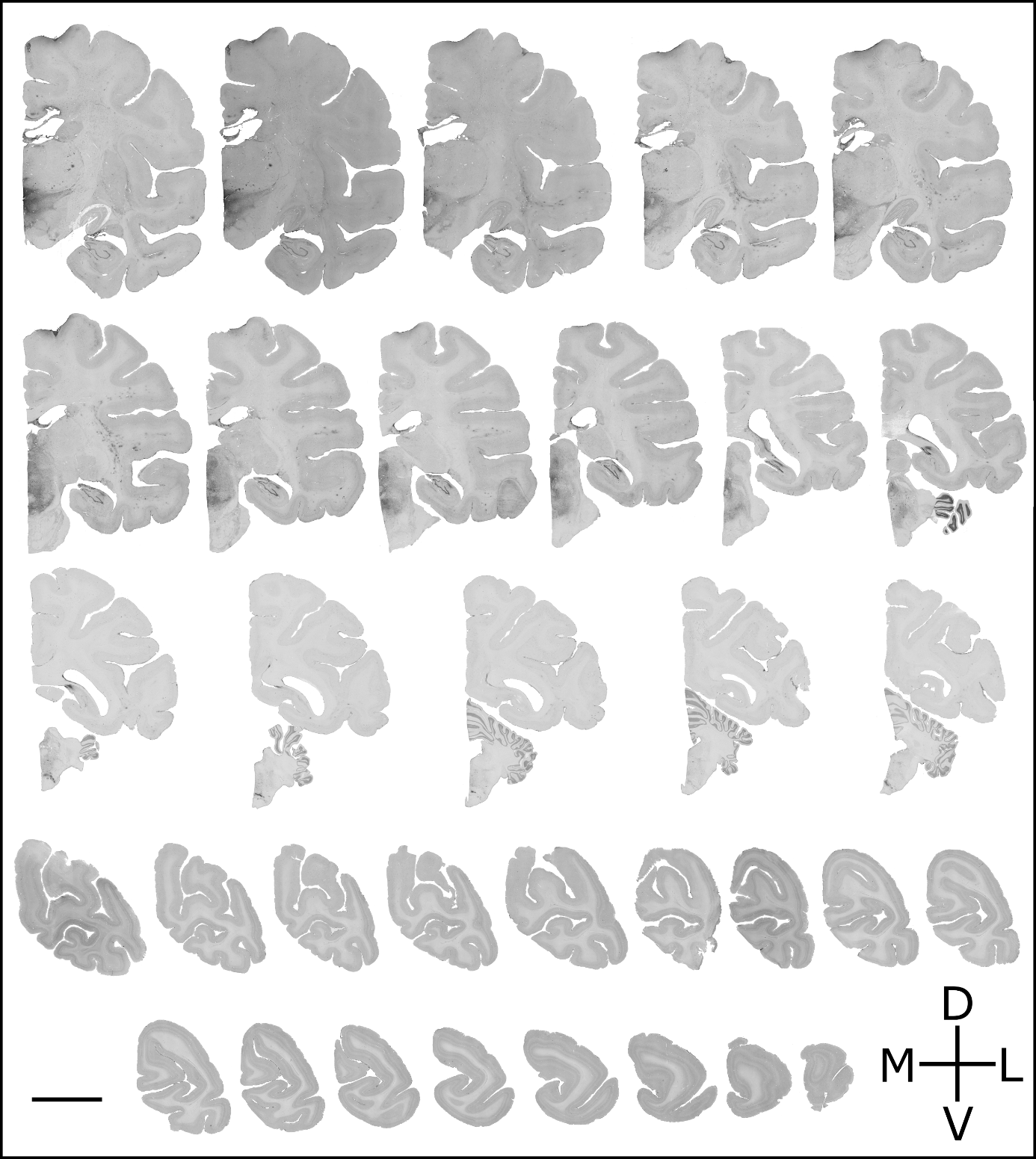


**Supplement 5: Overview of all IHC stained coronal sections of the hemisphere ipsilateral to the injection in M3.** Conventions follow those in Supplement 3; but presented coronal slices are from the ipsilateral hemisphere in M3.


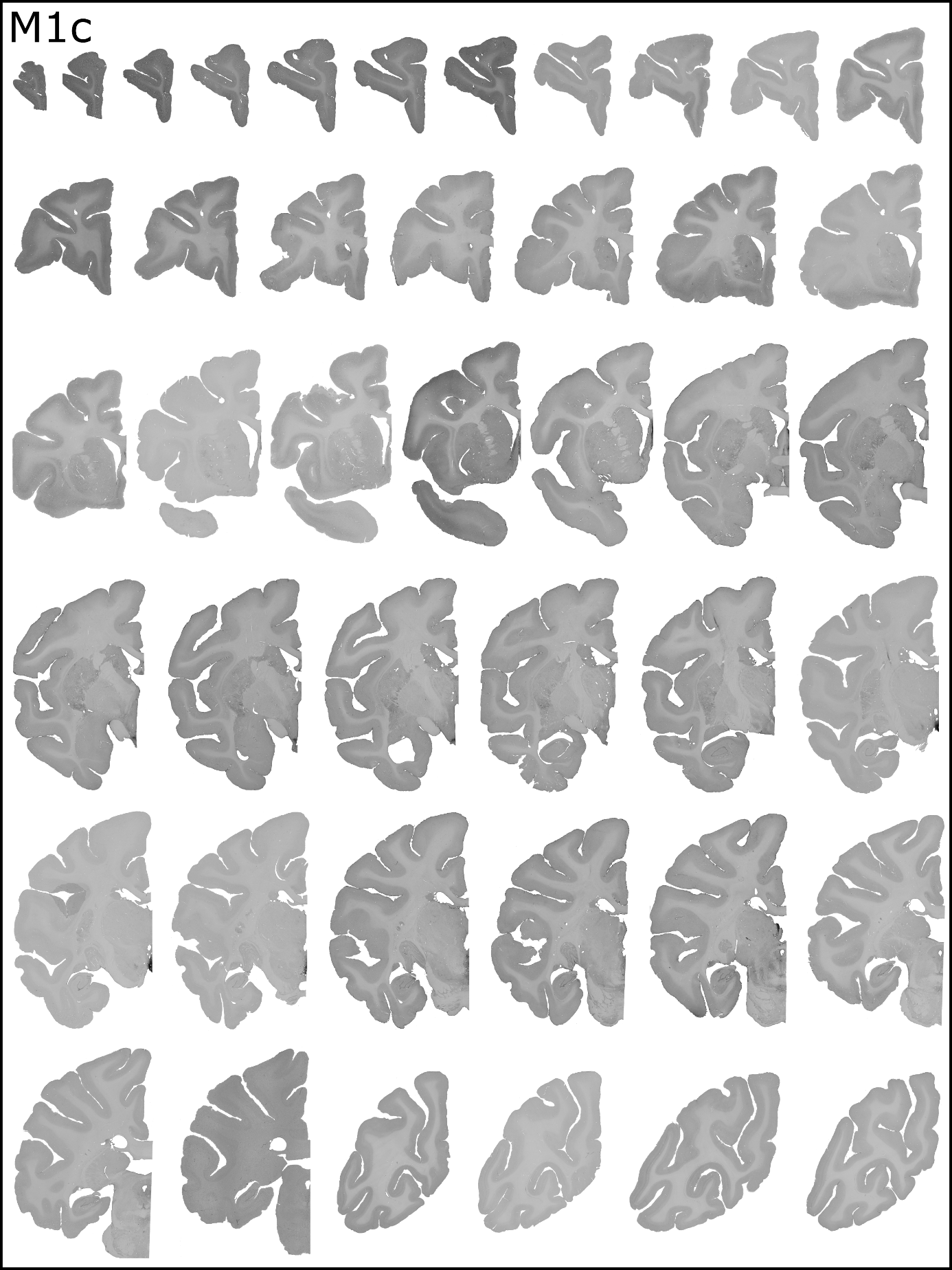


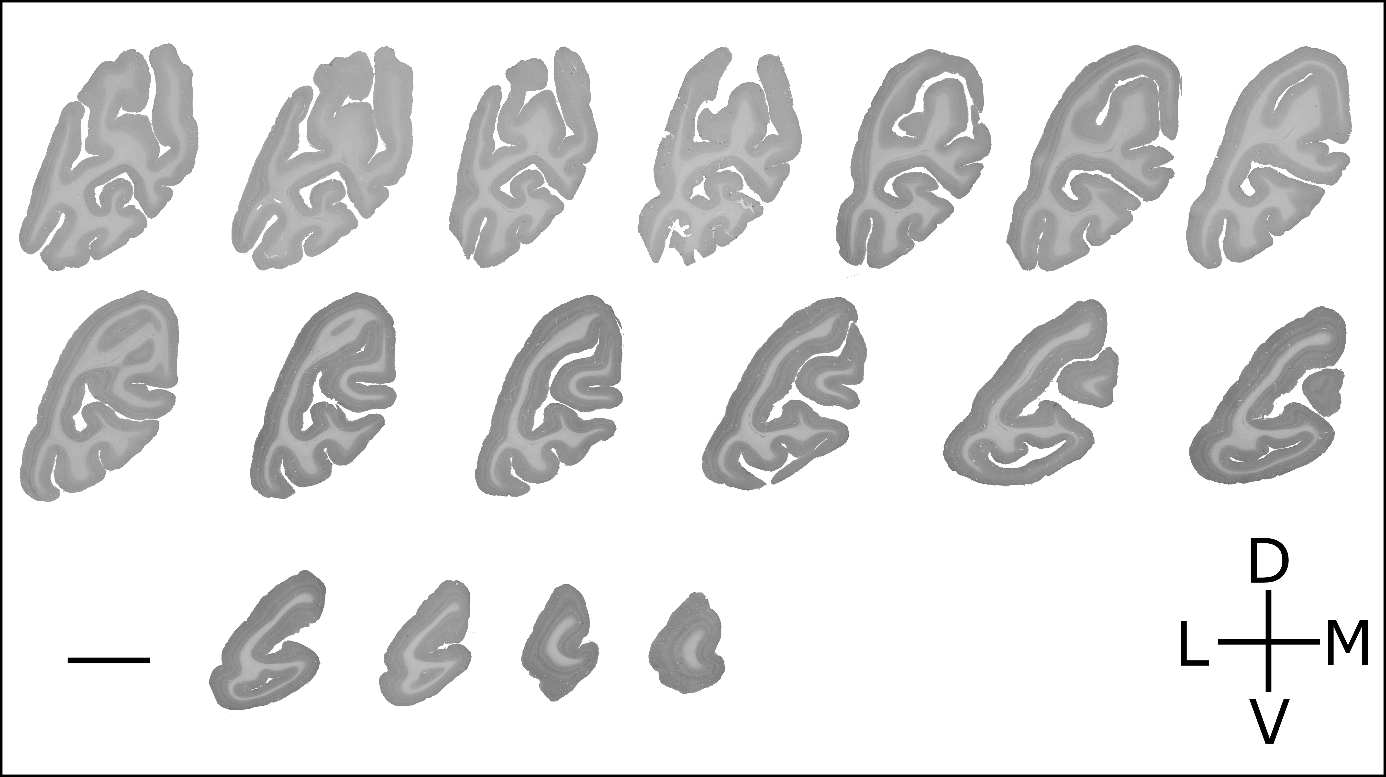


**Supplement 6: Overview of all IHC stained coronal sections of the hemisphere contralateral to the injection in M1.** Conventions follow those in Supplement 3; here we present coronal slices from the hemisphere contralateral to the injections in the same animal (M1).

**List of Abbreviations**

AAV adeno-associated virus

ACC anterior cingulate cortex

AIP anterior intraparietal area

AK auditory koniocortex

Amyg amygdala

AO anterior olfactory cortex

aT anterior thalamus

Aud auditory cortex

CA field CA of the hippocampus

Cd caudate nucleus

Cereb cerebellum

Cl claustrum

CT computed tomography

DA dopamine

DAB diaminobenzidine

Dg dentate gyrus

DPFC dorsal prefrontal cortex

ECL entorhinal cortex, caudal limited part

EGP external globus pallidus

EI entorhinal cortex, intermediate part

ELC entorhinal cortex, lateral part, caudal division

Ent entorhinal cortex

ER entorhinal cortex, rostral part

fMRI functional magnetic resonance imaging

fr fasciculus retroflexus

FST fundus of superior temporal sulcus

Gu gustatory cortex

Hipp hippocampus

IA agranular insula

ID dysgranular insula

IG granular insula

IGP internal globus pallidus

IHC immunohistochemistry

Ins insula

IPa intraparietal sulcus associated area in the superior temporal sulcus

IPar intraparietal cortex

IPro insular proisocortex

ITG inferior temporal gyrus

LGN lateral geniculate nucleus

LIPd lateral intraparietal area, dorsal part

LIPv lateral intraparietal area, ventral part

mdT medial dorsal thalamus

MGN medial geniculate nucleus

MIP medial intraparietal area

mml medial medullar lamina

MRI magnetic resonance imaging

MST medial superior temporal area

MTG medial temporal gyrus

NAc(c) nucleus accumbens (core)

NAc(s) nucleus accumbens (shell)

NB nucleus basalis

NGS normal goat serum

OFC orbitofrontal cortex

OPAI orbital periallocortex

OPro orbital proisocortex

PaAC para-auditory area, caudal part

PaAL para-auditory area, lateral part

PaAR para-auditory cortex, rostral part

PAG periaqueductal gray

PaIL parainsular area, lateral part

PaIM parainsular area, medial part

Par parietal cortex

PBS phosphate buffered saline

PCC posterior cingulate cortex

PE parietal area PE

PF parietal area PF

PFC prefrontal cortex

PG parietal area PG

PGa PG associated area of the superior temporal sulcus

PGM parietal area PG, medial part

Pir piriform cortex

ProK prokoniocortex

ProM promotor area

Pu putamen

Pul pulvinar nuclei

ReI retroinsular area

RFP red fluorescent protein

RRF retrorubral field

Rt reticular nucleus (prethalamus)

SII secondary somatosensory cortex

SNc substantia nigra, pars compacta

SNr substantia nigra, pars reticulata

Somato somatosensory cortex

ST superior temporal sulcus

STG superior temporal gyrus

STN subthalamic nucleus

Sub subiculum

TAa temporal area TAa

TE temporal area TE

TEa temporal area TEa

TEM temporal area TE, medial part

TEO temporal area TE, occipital part

TF temporal area TF

TFO temporal area TF, occipital part

TH temporal area TH

TH tyrosine hydroxylase

THO temporal area TH, occipital part

TL temporal area TL

TLR temporal area TL, rostral part

TO temporal-occipital cortex

TP temporal pole

TPO temporal-parietal-occipital

TPt temporoparietal area

V1 visual area 1

V2 visual area 2

V3A visual area 3A

V3D visual area 3, dorsal part

V3V visual area 3, ventral part

V4 visual area 4

V4V visual area 4, ventral part

V5 visual area 5

V6 visual area 6

V6A visual area 6A

Vg viral genome

VIP ventral intraparietal sulcus

Vis visual cortex

vIT ventral lateral thalamus

VLPFC ventral lateral prefrontal cortex

VP ventral pallidum

VTA-EM ventral tegmental area – electrical microstimulation
